# Development and evaluation of the content validity, practicability and feasibility of the Innovative dementia-oriented Assessment system for challenging behaviour in residents with dementia

**DOI:** 10.1186/s12913-017-2469-8

**Published:** 2017-08-14

**Authors:** Margareta Halek, Daniela Holle, Sabine Bartholomeyczik

**Affiliations:** 1 German Center for Neurodegenerative Diseases (DZNE), Stockumer Str. 12, 58453 Witten, Germany; 2 School of Nursing Science, Witten/Herdecke University, Witten, Germany

**Keywords:** Instrument development, Validity, Challenging behaviour, Dementia, Nursing home, Content validity index

## Abstract

**Background:**

One of the most difficult issues for care staff is the manifestation of challenging behaviour among residents with dementia. The first step in managing this type of behaviour is analysing its triggers. A structured assessment instrument can facilitate this process and may improve carers’ management of the situation. This paper describes the development of an instrument designed for this purpose and an evaluation of its content validity and its feasibility and practicability in nursing homes.

**Methods:**

The development process and evaluation of the content validity were based on Lynn’s methodology (1998). A literature review (steps 1 + 2) provided the theoretical framework for the instrument and for item formation. Ten experts (step 3) evaluated the first version of the instrument (the Innovative dementia-oriented Assessment (IdA®)) regarding its relevance, clarity, meaningfulness and completeness; content validity indices at the scale-level (S-CVI) and item-level (I-CVI) were calculated. Health care workers (step 4) evaluated the second version in a workshop. Finally, the instrument was introduced to 17 units in 11 nursing homes in a field study (step 5), and 60 care staff members assessed its practicability and feasibility.

**Results:**

The IdA® used the need-driven dementia-compromised behaviour (NDB) model as a theoretical framework. The literature review and expert-based panel supported the content validity of the IdA®. At the item level, 77% of the ratings had a CVI greater than or equal to 0.78. The majority of the question-ratings (84%, *n* = 154) and answer-ratings (69%, *n* = 122) showed valid results, with none below 0.50. The health care workers confirmed the understandability, completeness and plausibility of the IdA®. Steps 3 and 4 led to further item clarification. The carers in the study considered the instrument helpful for reflecting challenging behaviour and beneficial for the care of residents with dementia. Negative ratings referred to the time required and the lack of effect on residents´ behaviour.

**Conclusions:**

There was strong evidence supporting the content validity of the IdA®. Despite the substantial length and time requirement, the instrument was considered helpful for analysing challenging behaviour. Thus, further research on the psychometric qualities, implementation aspects and effectiveness of the IdA® in understanding challenging behaviour is needed.

**Electronic supplementary material:**

The online version of this article (doi:10.1186/s12913-017-2469-8) contains supplementary material, which is available to authorized users.

## Background

The process of caring for people with dementia in nursing homes is complicated by their manifestation of behavioural and psychological symptoms, also referred to as challenging behaviours [1]. Challenging behaviours occur throughout all phases of the disease [2] and are highly prevalent in nursing homes (82%) [3]. In contrast with loss of memory, cognitive decline, and functional disability, challenging behaviours are more difficult to manage and are often perceived to be a burden by care staff [4, 5].

Studies that investigate patients’ perspectives on the disease commonly consider challenging behaviours to be a way of coping with dementia rather than a behavioural problem [6]. Research has indicated that challenging behaviours often emerge from an incongruence between a person’s needs and the degree to which the environment fulfils those needs [7]. Thus, challenging behaviours can be explained as the best response a person can provide given the limitations imposed by dementia, his or her abilities and elements of the environment [8]. It is highly important that challenging behaviours are not taken for granted; rather, they should be explored by others to discover the possible triggers and causes of these behaviours [6].

Understanding the underlying causes of challenging behaviours is both demanding and complex. Assessment tools designed to systematically guide nursing staff through this process can facilitate these complex behavioural analyses [9]. These instruments enable an objective description and evaluation of challenging behaviours and can provide insight into their potential triggers and causes [10].

At the time of this study, existing assessment instruments (e.g., Behavioural Pathology in Alzheimer’s Disease (Behave-AD) [11], Neuropsychiatric Inventory-Nursing home version (NPI-NH) [12], Cohen-Mansfield-Agitation-Inventory (CMAI) [13], Challenging Behaviour Scale (CBS) [14]) include topographical information about the behaviour (nature, intensity, and frequency) and a measure of its consequences (stress, safety), but they do not comprise information about its possible triggers. These instruments had objectives other than understanding a person’s challenging behaviour. No instruments for use in nursing homes have been developed that capture all the information needed to systematically guide nursing and care staff through the complex process of understanding residents’ challenging behaviours using a biomedical and psychosocial definition of this behaviour. However, several structured approaches for the analysis of challenging behaviour within nursing homes have now been developed [15–20] and these approaches include assessment instruments, charts or tools for the analysis of challenging behaviour [21]. Few approaches also address a behavioural analysis in the community setting [22–25]. Due to a lack of detailed information on these documents, a thorough comparison of these instruments is not possible. These instruments appear to be broadly targeted and address different topics, although the determination of medical and physical causes of challenging behaviour is prominent in most assessment instruments [19, 21]. The assessment instruments often require the expertise of professionals other than nurses (e.g., nursing home physicians or psychologists) [16–19], or the assessment process is led by a psychologist with a multidisciplinary team [15]. This situation makes it more difficult or even impossible to transfer the assessment instrument to countries, like Germany, where psychologists or nursing home physicians are not employed in nursing homes. In Germany, the nurses are the professionals who are responsible for resident’s care. Additionally, these instruments have objectives other than understanding a person’s challenging behaviour.

To address these shortcomings, an assessment tool called the “Innovative dementia-oriented Assessment system (IdA®)” was developed and validated for use in nursing practice. The objectives of this assessment instrument were as follows: (1) it should comprehensively assess the aspects relevant to evaluating challenging behaviour such as its topography, consequences and need for action; (2) it should collect information that helps identify the triggers and causes and better understand the behaviour; (3) it should include information relevant to providing nursing care for people with dementia; and (4) it should be applicable by care staff.

This paper presents the (1) development process and the (2) content validity of the IdA® as well as the (3) results of its first feasibility and practicability tests.

## Methods

**Fig. 1:**
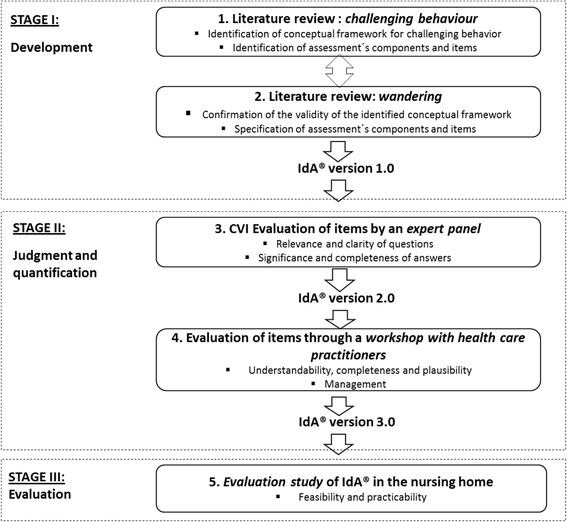
Stages of the development and validity and feasibility testing of the IdA®

For the *development* and *content validity* of the new instrument, the approach described by Lynn [26] was used. This approach advocates two stages, in which stage I (development) results in the generation of the instrument’s items and stage II evaluates the performance of the instrument’s items (judgement and quantification). In this study, these two stages were supplemented by a third stage (evaluation), in which the feasibility and practicability of the new instrument were tested within an evaluation study (Fig. 1).

### Stage I. Development

The development of the IdA® consisted of two comprehensive literature reviews. The first review aimed to identify a conceptual framework for challenging behaviour and to identify the assessment’s components and items [26–28]. The second literature review focused on the specific behaviour of wandering and aimed to confirm the validity of the conceptual framework and of the specific components and items found in the first review. Both reviews were conducted in parallel and used an iterative process.

The first literature review was guided by following research questions:

1. Which conceptual framework is most suitable for assessing challenging behaviour and its causes in people with dementia who are living in nursing homes?
2. Which components of the conceptual framework are the most important for establishing the instrument’s dimensions?
3. Which items are the most important for representing/assessing the instrument’s dimensions?

The following research question guided the second literature search:

4. Are additional dimensions or items needed when considering a specific challenging behaviour?

The search process for both reviews has been described elsewhere [29].

Based on the results of both literature reviews, a first draft of the instrument and its items was developed (IdA® version 1.0).

### Stage II. Judgement and quantification

The Judgement and Quantification phase included two evaluations. The first assessment consisted of an expert panel that focused on the relevance and clarity of the questions as well as the significance and completeness of responses in the IdA®. The second evaluation comprised a workshop with health care workers that focused on the understandability, completeness, plausibility and management of the IdA®.

#### 1st evaluation: Expert panel

Experts in the expert panel were defined as individuals who worked in practice as a practitioner or in science in the field of dementia care, i.e., the criteria included being a nurse or being very familiar with nursing work and having an understanding of the field of assessment instruments. We focused mainly on nurses since nurses in Germany are responsible for the nursing process and thus for recognition and management of challenging behaviour. Fifteen experts were identified and contacted.

#### Measurement of content validity

As the IdA® was composed of questions and answers, a content validity index was calculated for both.

Each question was evaluated by rating a) its relevance to the instrument’s aim and b) its understandability/clarity. Each answer was assessed regarding its c) comprehensiveness/completeness and d) meaningfulness/significance for the related question. The four attributes (a-d) of the questions and answers were rated on a 4-point scale (1 = not relevant/not clear/incomplete/meaningless; 4 = highly relevant/clear/complete/meaningful) [26, 30]. In addition, the experts were asked to evaluate whether the items covered all important aspects or if there were missing components. The experts could also comment on every item.

The experts in the first expert panel also completed a questionnaire about their demographics and expertise.

#### Measurement of the content validity index

A content validity index was calculated both at the item level (I-CVI) and scale level (S-CVI) for all four attributes [26, 31]. The I-CVI was calculated as the number of experts providing a score of 3 or 4 divided by the total number of experts [26]. With more than 5 experts, the I-CVI should not be lower than 0.78 [31].

To calculate the S-CVI, two different indices were calculated: 1) the proportion of the items on one scale that the experts scored as valid (ratings of 3 or 4) (universal agreement by experts = S-CVI/UA) and 2) the average proportion of the items on one scale rated 3 or 4 (average agreement by experts = S-CVI/Ave) [31]. The S-CVI/UA is sensitive to the number of experts: the more experts are included, the greater the possibility of generating a low S-CVI. The S-CVI/Ave is more liberal and is preferred by Polit and Beck [31]; however, they recommend presenting both indices. The acceptable standard for the S-CVI/UA and the S-CVI-Ave is 0.8 [30, 31]; values up to 0.9 indicate an excellent average [21].

Additionally, a modified Kappa index was computed to estimate the I-CVI [32, 33]. The modified Kappa (k*) is an index of agreement among experts that indicates beyond chance that the item is relevant, clear, or another characteristic of interest [32]. We used the formula suggested by Polit et al. [32] (Table 5). The standards recommended by Fleiss [34] and Cicchetti and Sparrow [35] were used to interpret k*.

The results of the expert panel contributed to the second version of the instrument (IdA® 2.0).

#### 2nd evaluation: Workshop with health care workers

According to Lynn [26], the same experts should re-evaluate the modified version of an instrument. Due to the comprehensiveness of the instrument and the limited resources of the first expert panel, a second round of evaluations with the same experts was not possible. Therefore, a second evaluation was organized with other experts, in which key persons from health care practice were invited to participate in a workshop. The nursing homes were free to select the key persons; the only inclusion criteria were that the persons were responsible for implementing the IdA® in this study, e.g., organizing the application of the IdA® and/or using it within the planned study, and that they had experience caring for people with dementia.

During the workshop, which lasted for several hours, the modified version of the IdA® (version 2.0) was introduced, and the objectives were explained. Subsequently, the experts were asked to assess the understandability, plausibility and completeness of the items. The discussion and suggested modifications were recorded, resulting in a further revised version of the IdA® (version 3.0).

### Stage III: Evaluation

The third stage aimed to evaluate the feasibility of the IdA®, its usefulness in managing challenging behaviour and its practicability in daily work through an evaluation study. Data from a questionnaire administered in a before-after study were used to evaluate the feasibility and relevance of the IdA® in nursing practice.

### Sample

A convenience sample of 11 nursing homes with 17 units participated in the evaluation study. Twelve were traditional care units and 5 were specialized care units for residents with dementia. All units had structures that enabled the use of the IdA® within a team context (e.g., team meetings, case conferences, nursing rounds). All care staff members were invited to the survey.

### Application of the IdA®

In this study, the IdA® was expected to be used for 6 weeks with a minimum of 3 residents in one care unit (traditional and/or specialized). The IdA® was introduced to key persons on the units’ care staff team, who received a comprehensive one-day training that included lectures about dementia and challenging behaviour, guidelines about managing challenging behaviour and the IdA® dimensions and items. The remaining care staff team members received an one-hour training on how to use the IdA®.

The IdA® should be used in the study as a team instrument for care staff: the collection of information, the formulation of hypotheses about the causes and triggers of the behaviour and the development of the intervention plan should be conducted within the care staff team (e.g., team meetings, case conferences). Discussions and negotiation are necessary for an appropriate use of the IdA®.

### Data collection and analysis

To evaluate the feasibility and relevance of the IdA®, self-developed questionnaires were used. The questionnaires included ratings about its structural aspects (15 items: clarity, meaningfulness, completeness, length, comprehensibility and background information required to use the IdA®); its benefit for daily care (8 items: professionalism and use of the IdA® as a memory aid, communication aid, planning aid, evaluation aid and information tool); carers’ experiences with the IdA® and an overall rating of the IdA®. An open question format was used to ask about missing information, problems with comprehension and other aspects. Finally, the caregivers were asked whether the IdA® supported them in decision making about residents’ challenging behaviour. The data were analysed using descriptive statistics (proportion and mean).

## Results

### Stage I: Development

The literature review identified a few models or approaches that aimed to explain the development of challenging behaviour in people with dementia (the multidimensional model [36], Progressively Lowered Stress Threshold model [37], Treatment Routes for Exploring Agitation (TREA) [38], Need driven dementia-compromised behaviour model (NDB model) [39], Serial Trial Intervention (STI) [40], Comprehensive model of behaviour [41], Antecedents-Behavior-Consequences (ABC)-approach [31]. In addition to the above mentioned approaches, further dementia-relevant psychosocial models exist [42]. These comprehensive and multidimensional models did not focus solely on behaviour but incorporated assumptions about the development of challenging behaviour in dementia.

The complexity of the behaviour and the influence of multiple determinants call for an explanation model that considers all aspects mentioned in the different approaches [33].

From the nursing perspective, the NDB model fulfilled all of the requirements for this study. It integrated the bio-medical and psychosocial perspective and provided a literature-based, comprehensible and comprehensive framework for assessing the causes of challenging behaviour. The origin of the model was in nursing science [39].

In this model, the causes of challenging behaviour are the unmet needs of people with dementia. They result from the increasing inability of these people to care for themselves, to interpret their needs as such and to communicate them in an understandable manner. The interaction between proximal (physiological and psychological aspects, characteristics of the environment) and background (e.g., neurological factors, cognitive abilities, health status, physical functioning, premorbid personality) factors is hypothesized to lead to the development of challenging behaviour. Background factors are stable characteristics of the person, with low changes over time, and act as risk factors and mediators for the influence of proximal factors on the development of challenging behaviour. Proximal factors are changeable aspects of the person with dementia or the direct environment and can directly produce challenging behaviour [39, 43]. For example, a person has a feeling of fear or experiences pain (proximal factors). The impairments in verbal communication (background factor) and a sensitive and fearful personality (background factor) determine how (through repetitive questioning “Hallo, Help, Hallo, Help”), in which situation, and how rapidly the feeling of anxiety evolves. The interplay between background and proximal factors determines how the person’s needs are communicated, expressed or satisfied. The literature on wandering did not produce new dimensions or aspects that were not covered by the NDB model. The results of the literature review provided substantial information about the priorities of the model and how to define and assess different triggers.

Additionally, other aspects were identified as relevant for documenting the behaviour itself and for deciding whether the behaviour was challenging or problematic and whether it required further consideration. These factors included a description of the type of behaviour, its quantity, context, burden and risk potential. The review showed a large number of scales and measurements, but none of them included all of the issues mentioned above. Thus, an assessment of all attributes of challenging behaviour was developed. The Cohen-Mansfield agitation model [44, 45] was identified as the one most commonly used in research to describe challenging behaviour. The underlying categorization of behaviour types established the theoretical foundation for the development of items in the IdA®. Apathy was added because the Cohens-Mansfield agitation model did not focus on this type of behaviour and apathy is one of the most prevalent challenging behaviours in nursing homes [3].

The first version (1.0) of the instrument consisted of 6 domains within 2 main parts: assessment of behaviour (part 1) and assessment of the causes and triggers of the behaviour (part 2). The behavioural assessment included 18 items, and part 2 was composed of 5 domains (health status & independence in everyday life, communication, personality & life style, moods & emotions, and environmental influences) with 8 dimensions (cognitive status, physical health status, physical discomfort, independence in everyday life, communication, personality and lifestyle before the onset of dementia, mood & emotions, and environmental influences). In total, 93 items were developed (Table 1). The items had different formats: closed-ended and open-ended. Examples are shown in Table 2.

**Table 1:** Different versions of the IdA®; results from stages I and II

Domains	Version 1.0 (93 items) Prior to the expert evaluation as a result of Stage I, steps 1 & 2		Version 2.0 (72 items) after expert evaluation as a result of Stage II, step 3		Version 3.0 (89 items)* After evaluation by key persons in Stage II, step 4	
	Dimensions	Number of items	Dimensions	Number of items	Dimensions	Number of items
PART 1						
1. Assessment of the behaviour	Challenging behaviour	18	Challenging behaviour	14	Challenging behaviour	14
PART 2						
2. Health status & independence in everyday life	Cognitive status	7	Cognitive status	11	Cognitive status	11
	Physical health status	5	Physical health status and discomfort	11	Physical health status and discomfort	10
	Physical discomfort	13				
	Independence in everyday life	17	Independence in everyday life	1	Independence in everyday life	2
3. Communication	Communication	6	Communication	6	Communication	9
4. Personality & lifestyle before dementia	Personality & lifestyle before the onset of dementia	8	Personality & lifestyle before the onset of dementia	8	Personality & lifestyle before the onset of dementia	8
5. Moods & emotions	Moods & emotions	7	Moods & emotions	10	Moods & emotions	10
6. Environmental influences	Environmental influences	11	Environmental influences	11	Environmental influences	11
PART 3						
7. Summary					Overview and summary	14

**Table 2:**
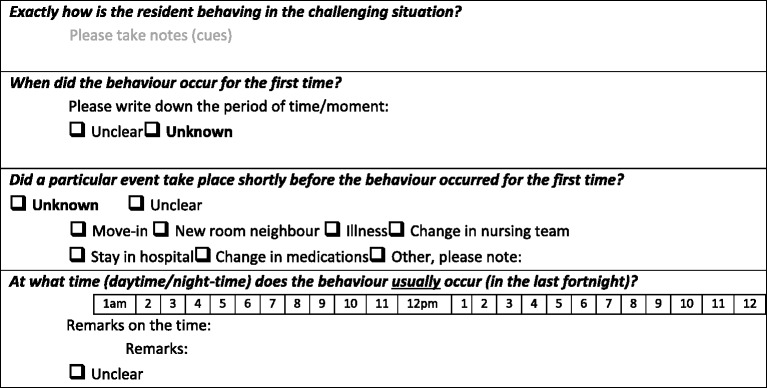
Examples of items in version 1.0

### Stage 2: Judgement and quantification

#### 1st evaluation: Expert panel

##### Sample

Ten out of 15 experts who were contacted completed the evaluation forms. The only reason for non-completion was a lack of time. The data confirmed that these persons had the required expertise to evaluate the instrument (Table 3). The experts were from a broad spectrum of disciplines; most of them had a double qualification, for example, as a nurse and a gerontologist. Almost all the experts had additional qualification in dementia-relevant aspects. All the experts had longstanding experience in the dementia field. Four of the experts were members of the group of experts who developed the German guidelines for challenging behaviours of residents living in nursing homes [46].

**Table 3:** Sample characteristics of the expert panel

Characteristics of expert panel, *N* = 10	
Gender	3 men, 7 women
Academic Disciplines (some with more than one degree)	4 nursing/nursing science, 1 psychiatry/psychotherapy, 2 pedagogy/gerontology, 1 psycho-gerontology, 1 philosophy, 1 literature, 1 economics, 1 cultural studies
Vocational training	2 geriatric nurses, 5 nurses
Position	3 employed by the university/development and research institution (scientist, lecturer), 2 in leading positions in nursing homes, 2 working in quality development, 2 consultants in dementia care, 1 senior physician/freelancer
Additional dementia-related training	2 validation, dementia care mapping; 3 geriatric psychiatry/gerontology; 2 cognitive training, communication, organizational development and quality management; 1 palliative medicine
General work experience	On average, 20 years (min 4; max 35)
Work experience in dementia care	On average, 12 years (min 3; max 25)
Practice with people with dementia:	
Care of people with dementia	9 experts
Assessment instruments/process documentation	9 experts
Theoretical knowledge about:	
Care of people with dementia	10 experts
Process documentation	10 experts
Assessment instruments in nursing	9 experts
Research experience:	
In field of dementia	4 experts
With assessment instruments/process documentation	3 experts
Self-assessment of own expertise in the following:	
Dementia care	Median 3^a^
Nursing assessment/documentation	Median 2^a^

^a^Ranked between 0 = no expertise and 3 = comprehensive expertise

##### Average S-CVI

The S-CVI was calculated for all 9 dimensions, resulting in 36 CVI indices for each calculation method (average and universal agreement).

The S-CVI/Ave ranged from 0.64 to 1.00. Six (16.7%) of the 36 indices were lower than 0.80, and 10 indices (28%) were lower than 0.90. The majority of the dimensions indicated content validity according to both cut-offs (83% and 72% for 0.80 and 0.90, respectively). The questions showed a better performance than the answers. All S-CVI/UA values except one were below the acceptable minimum of 0.80 (Table 4).

**Table 4:** S-CVI for the IdA® Version 1.0

Dimensions of IdA®, 1.0	Relevance of Questions S-CVI/Ave	Clarity of Questions S-CVI/Ave	Significance of Answers S-CVI/Ave	Completeness of Answers S-CVI/Ave
1. Assessment of behaviour	0.92	0.87	0.87	0.83
2. Cognitive status	0.95	0.85	0.83	0.74
3. Physical health status	1.00	0.90	0.88	0.79
4. Physical discomfort	0.92	0.81	0.82	0.77
5. Independence in everyday life	0.81	0.85	0.72	0.72
6. Communication	0.93	0.91	0.81	0.83
7. Personality & lifestyle before the onset of dementia	0.95	0.92	0.87	0.90
8. Mood & emotions	0.87	0.64	0.80	0.80
9. Environmental influences	0.85	0.81	0.80	0.76

Questions in the independence in everyday life dimension received the lowest relevance scores for S-CVI/Ave (S-CVI/Ave 0.81), followed by the environmental influences (S-CVI/Ave 0.85) and mood & emotions (S-CVI/Ave 0.87). The best score for the relevance of questions was for the dimension physical health status at 1.0, followed by cognitive status and personality & lifestyle before the onset of dementia (both S-CVI/Ave 0.95) (Table 4).

The average S-CVI for understandability ranged between 0.64 and 0.92. The questions on mood & emotions were the least understood (S-CVI/Ave 0.64). Personality & lifestyle before the onset of dementia (S-CVI/Ave 0.92) as well as communication (S-CVI/Ave 0.91) appeared to be best understood (Table 4).

The scores for meaningfulness of the answers ranged from 0.72 to 0.88. The cognitive status dimension received the fewest negative values (S-CVI/Ave 0.88) followed by the assessment of behaviour (S-CVI/Ave 0.87). The weakest dimension regarding the meaningfulness of answers was independence in everyday life (S-CVI/Ave 0.72) (Table 4).

The answers for 5 of 9 subject areas appeared to be incomplete (S-CVI/Ave below 0.8). S-CVI/Ave varied between 0.72 and 0.9. The dimension personality & lifestyle before the onset of dementia had the highest scores, and the subject areas independence in everyday life, cognitive status and environmental influences received the lowest scores.

##### Item -CVI

The experts scored 93 items regarding 4 attributes, two for each set of questions and answers, and thus a total of 360 CVI indices and k* were calculated. On the item level, 77**%** (276) of the ratings had a CVI greater than or equal to 0.78. The majority of the question-ratings (84%, *n* = 154) and answer-ratings (69%, *n* = 122) showed valid results. None of the ratings were below 0.50, which would indicate rejection of the item. Fifty-one complete items (question plus answer options) showed an I-CVI value less than 0.78 and k* less than 0.74 for at least one of the 4 rated attributes. Only 6 had I-CVI values ≤0.5 and k* < 0.4. The results for each item are shown in Tables 5, 6, 7, 8, 9, 10, 11, 12 and 13.

**Table 5:** Content validity of the dimension “Assessment of behaviour”

		Relevance of the questions						Clarity of the questions						Meaningfulness of the answers						Completeness of the answers					
No	Content of the items	Number of experts	Number of ratings of 3 or 4	I-CVI^a^	p_c_^b^	k* ^c^	Evaluation^d^	Number of experts	Number of ratings of 3 or 4	I-CVI^a^	p_c_^b^	k* ^c^	Evaluation^d^	Number of experts	Number of ratings of 3 or 4	I-CVI^a^	p_c_^b^	k* ^c^	Evaluation^d^	Number of experts	Number of ratings of 3 or 4	I-CVI^a^	p_c_^b^	k* ^c^	Evaluation^c^
1	Type of behaviour	10	10	1.00	0.001	1.00	****	10	8	0.80	0.044	0.79	****	9	8	0.89	0.018	0.89	****	9	7	0.78	0.070	0.76	****
2	Description of behaviour	10	10	1.00	0.001	1.00	****	10	9	0.90	0.010	0.90	****	9	8	0.89	0.018	0.89	****	8	6	0.75	0.109	0.72	***
3	First occurrence of behaviour	10	9	0.90	0.010	0.90	****	10	10	1.00	0.001	1.00	****	9	9	1.00	0.002	1.00	****	8	8	1.00	0.004	1.00	****
4	Event before the occurrence of the behaviour	10	10	1.00	0.001	1.00	****	10	10	1.00	0.001	1.00	****	9	8	0.89	0.018	0.89	****	8	7	0.88	0.031	0.87	****
5	Time at which the behaviour occurs	10	10	1.00	0.001	1.00	****	10	9	0.90	0.010	0.90	****	9	9	1.00	0.002	1.00	****	9	6	0.67	0.164	0.60	***
6	Duration of the behaviour	10	10	1.00	0.001	1.00	****	10	9	0.90	0.010	0.90	****	9	7	0.78	0.070	0.76	****	9	7	0.78	0.070	0.76	****
7	Frequency of the behaviour	10	10	1.00	0.001	1...00	****	10	10	1.00	0.001	1.00	****	8	7	0.88	0.031	0.87	****	9	9	1.00	0.002	1.00	****
8	Area in which the behaviour occurs	9	8	0.89	0.018	0.89	****	9	9	1.00	0.002	1.00	****	9	8	0.89	0.018	0.89	****	9	7	0.78	0.070	0.76	****
9	Person present when the behaviour occurs	10	8	0.80	0.044	0.79	****	10	9	0.90	0.010	0.90	****	9	7	0.78	0.070	0.76	****	9	7	0.78	0.070	0.76	****
10	Events directly before the behaviour	10	10	1.00	0.001	1.00	****	10	9	0.90	0.010	0.90	****	9	8	0.89	0.018	0.89	****	8	6	0.75	0.109	0.72	***
11	Events directly after the behaviour	10	10	1.00	0.001	1.00	****	10	9	0.90	0.010	0.90	****	9	9	1.00	0.002	1.00	****	8	8	1.00	0.004	1.00	****
12	Helpful interventions	10	8	0.80	0.044	0.79	****	10	5	0.50	0.246	0.34	*	9	7	0.78	0.070	0.76	****	8	7	0.88	0.031	0.87	****
13	Behaviour unpleasant for the resident	10	8	0.80	0.044	0.79	****	10	9	0.90	0.010	0.90	****	9	8	0.89	0.018	0.89	****	9	6	0.67	0.164	0.60	***
14	Danger for the person with dementia	10	10	1.00	0.001	1.00	****	10	9	0.90	0.010	0.90	****	9	9	1.00	0.002	1.00	****	9	7	0.78	0.070	0.76	****
15	Behaviour unpleasant for the resident or for other residents	10	8	0.80	0.044	0.79	****	10	9	0.90	0.010	0.90	****	8	6	0.75	0.109	0.72	***	9	9	1.00	0.002	1.00	****
16	Danger for other residents	10	10	1.00	0.001	1.00	****	11	9	0.82	0.027	0.81	****	9	8	0.89	0.018	0.89	****	9	7	0.78	0.070	0.76	****
17	Impact on carers	10	8	0.80	0.044	0.79	****	10	6	0.60	0.205	0.50	**	9	8	0.89	0.018	0.89	****	9	7	0.78	0.070	0.76	****
18	Danger for carers	10	8	0.80	0.044	0.79	****	8	7	0.88	0.031	0.87	****	9	9	1.00	0.002	1.00	****	10	9	0.90	0.010	0.90	****
		S-CVI/AVE 0.92						S-CVI/AVE 0.87			****			S-CVI/AVE 0.87						S-CVI/AVE 0.83					
		S-CVI/UA 0.56						S-CVI/UA 0.22						S-CVI/UA 0.28						S-CVI/UA 0.17					

^a^ I-CVI (content validity index) = number of experts providing a rating of 3 or 4/number of experts

^b^ p_c_ (probability of chance occurrence) = [N!/A!(N-A)!] × 0.5^N,^ N = number of experts; A = number of experts agreeing on a rating of 3 or 4

^c^ k* (modified kappa) = (I-CVI-p_c_)(1-p_c_)

^d^ Evaluation criteria for the level of content validity: relationship between I-CVI and k*; excellent validity = I-CVI ≥ 0.78 and k* >0.74 (****); good validity I-CVI < 0.78 and ≥ 0.60 and k* ≤ 0.74 (***); fair validity I-CVI < 0.6 and ≥ 0.40 and k* ≤ 0.59 (**); and poor validity I-CVI < 0.4 and k* <0.40 (*)

**Table 6:** Content validity of the dimension “Cognition status”

		Relevance of the questions						Clarity of the questions						Meaningfulness of the answers						Completeness of the answers					
No	Content of the item	Number of experts	Number of ratings of 3 or 4	I-CVI^a^	p_c_^b^	k* ^c^	Evaluation^d^	Number of experts	Number of ratings of 3 or 4	I-CVI^a^	p_c_^b^	k* ^c^	Evaluation^d^	Number of experts	Number of ratings of 3 or 4	I-CVI^a^	p_c_^b^	k* ^c^	Evaluation^ad^	Number of experts	Number of ratings of 3 or 4	I-CVI^a^	p_c_^b^	k* ^c^	Evaluation^d^
1	Diagnosis of dementia	10	10	1.00	0.001	1.00	****	10	9	0.90	0.010	0.90	****	10	8	0.80	0.044	0.79	****	9	7	0.78	0.070	0.76	****
2	Type of dementia	10	10	1.00	0.001	1.00	****	10	10	1.00	0.001	1.00	***	10	8	0.80	0.044	0.79	****	10	7	0.70	0.117	0.66	***
3	Dementia stage	10	10	1.00	0.001	1.00	****	10	8	0.80	0.044	0.79	****	10	6	0.60	0.205	0.50	**	8	6	0.75	0.109	0.72	***
4	Memory functions	8	6	0.75	0.109	0.72	***	8	7	0.88	0.031	0.87	****	9	9	1.00	0.002	1.00	****	9	6	0.67	0.164	0.60	***
5	Long-term memory	10	9	0.90	0.010	0.90	****	10	7	0.70	0.117	0.66	***	9	9	1 00	0.002	1.00	****	7	6	0.86	0.055	0.85	****
6	Short-time memory	10	10	1.00	0.001	1.00	****	10	8	0.80	0.044	0.79	****	10	8	0.80	0.044	0.79	****	9	6	0.67	0.164	0.60	***
7	Orientation	9	9	1.00	0.002	1.00	****	10	9	0.90	0.010	0.90	****	10	8	0.80	0.044	0.79	****	9	6	0.67	0.164	0.60	***
		S-CVI/AVE 0.95						S-CVI/AVE 0.85						S-CVI/AVE 0.83						S-CVI/AVE 0.74					
		S-CVI/UA 0.71						S-CVI/UA 0.14						S-CVI/UA 0.33						S-CVI/UA 0.00					

^a^ I-CVI (content validity index) = number of experts providing a rating of 3 or 4/number of experts

^b^ p_c_ (probability of chance occurrence) = [N!/A!(N-A)!] × 0.5^N,^ N = number of experts; A = number of experts agreeing on a rating of 3 or 4

^c^ k* (modified kappa) = (I-CVI-p_c_)(1-p_c_)

^d^ Evaluation criteria for level of content validity: relationship between I-CVI and k*; excellent validity = I-CVI ≥ 0.78 and k* >0.74 (****); good validity I-CVI < 0.78 and ≥ 0.60 andk* ≤0.74 (***); fair validity I-CVI < 0.6 and ≥ 0.40 and k* ≤0.59 (**); poor validity I-CVI < 0.4 and k* <0.40 (*)

**Table 7:** Content validity of the dimension “Physical health status”

		Relevance of the questions						Clarity of the questions						Meaningfulness of the answers						Completeness of the answers					
No	Content of the items	Number of experts	Number of ratings of 3 or 4	I-CVI^a^	p_c_^b^	k* ^c^	Evaluation^d^	Number of experts	Number of ratings of 3 or 4	I-CVI^a^	p_c_^b^	k* ^c^	Evaluation^d^	Number of experts	Number of ratings of 3 or 4	I-CVI^a^	p_c_^b^	k* ^c^	Evaluation^ad^	Number of experts	Number of ratings of 3 or 4	I-CVI^a^	p_c_^b^	k* ^c^	Evaluation^d^
1	Physical impairment	9	9	1.0	0.002	1.0	****	9	8	0.89	0.018	0.89	****	-	-	-	-	-	-	-	-	-	-	-	-
2	Mobility-related impairment	10	10	1.0	0.001	1.0	****	10	7	0.70	0.117	0.66	***	10	9	0.90	0.010	0.90	****	10	7	0.70	0.117	0.66	***
3	Food intake	10	10	1.0	0.001	1.0	****	10	10	1.00	0.001	1.00	****	10	9	0.90	0.010	0.90	****	9	7	0.78	0.070	0.76	****
4	Limitations with bowel or bladder function	10	10	1.0	0.001	1.0	****	10	10	1.00	0.001	1.00	****	10	8	0.80	0.176	0.76	****	9	8	0.89	0.018	0.89	****
5	Other physical limitations	10	10	1.0	0.001	1.0	****	10	9	0.90	0.010	0.90	****	10	9	0.90	0.010	0.90	****	10	8	0.80	0.044	0.79	****
		S-CVI/AVE 1.0						S-CVI/AVE 0.90						S-CVI/AVE 0.88						S-CVI/AVE 0.79					
		S-CVI/UA 1.0						S-CVI/UA 0.40						S-CVI/UA 0.00						S-CVI/UA 0.00					

^a^ I-CVI (content validity index) = number of experts providing a rating of 3 or 4/number of experts

^b^ p_c_ (probability of chance occurrence) = [N!/A!(N-A)!] × 0.5^N,^ N = number of experts; A = number of experts agreeing on a rating of 3 or 4

^c^ k* (modified kappa) = (I-CVI-p_c_)(1-p_c_)

^d^ Evaluation criteria for level of content validity: relationship between I-CVI and k*; excellent validity = I-CVI ≥ 0.78 and k* >0.74 (****); good validity I-CVI < 0.78 and ≥ 0.60 and k* ≤ 0.74 (***); fair validity I-CVI < 0.6 and ≥ 0.40 and  k* ≤0.59 (**); poor validity I-CVI < 0.4 and k* <0.40 (*)

**Table 8:** Content validity of the dimension “Independence in everyday life”

		Relevance of the questions						Clarity of the questions						Meaningfulness of the answers						Completeness of the answers					
No	Content of the items	Number of experts	Number of ratings of 3 or 4	I-CVI^a^	p_c_^b^	k* ^c^	Evaluation^d^	Number of experts	Number of ratings of 3 or 4	I-CVI^a^	p_c_^b^	k* ^c^	Evaluation^d^	Number of experts	Number of ratings of 3 or 4	I-CVI^a^	p_c_^b^	k* ^c^	Evaluation^d^	Number of experts	Number of ratings of 3 or 4	I-CVI^a^	p_c_^b^	k* ^c^	Evaluation^d^
1	Dependence in movement	10	9	0.90	0.010	0.90	****	10	10	1.00	0.001	1.00	****	10	9	0.90	0.010	0.90	****	10	9	0.90	0.010	0.90	-
2	Resources in movement	10	9	0.90	0.010	0.90	****	10	9	0.90	0.010	0.90	****	10	9	0.90	0.010	0.90	****	10	9	0.90	0.010	0.90	***
3	Dependence in personal hygiene	10	7	0.70	0.117	0.66	****	10	10	1.00	0.001	1.00	****	10	7	0.70	0.117	0.66	***	10	7	0.70	0.117	0.66	****
4	Resources in personal hygiene	10	8	0.80	0.044	0.79	****	10	10	1.00	0.001	1.00	****	10	7	0.70	0.117	0.66	****	10	7	0.70	0.117	0.66	****
5	Dependence in dressing	10	8	0.80	0.044	0.79	****	10	9	0.90	0.010	0.90	****	10	8	0.80	0.044	0.79	****	10	8	0.80	0.044	0.79	***
6	Resources in dressing	10	8	0.80	0.044	0.79	****	10	8	0.80	0.044	0.79	****	10	7	0.70	0.117	0.66	***	10	7	0.70	0.117	0.66	***
7	Dependence in nutrition	10	9	0.90	0.010	0.90	****	10	9	0.90	0.010	0.90	****	10	9	0.90	0.010	0.90	****	10	9	0.90	0.010	0.90	****
8	Resources in nutrition	9	8	0.89	0.018	0.89	****	10	9	0.90	0.010	0.90	****	10	8	0.80	0.044	0.79	****	10	8	0.80	0.044	0.79	****
9	Dependence in elimination	10	10	1.00	0.001	1.00	***	8	7	0.88	0.031	0.87	****	10	9	0.90	0.010	0.90	****	10	9	0.90	0.010	0.90	****
10	Resources in elimination	10	10	1.00	0.001	1.00	****	10	10	1.00	0.001	1.00	****	10	8	0.80	0.044	0.79	****	10	8	0.80	0.044	0.79	****
11	Dependence in leisure time activities	10	7	0.70	0.117	0.66	***	10	6	0.60	0.205	0.50	**	10	6	0.60	0.205	0.50	**	10	6	0.60	0.205	0.50	**
12	Resources in leisure time	10	7	0.70	0.117	0.66	***	10	8	0.80	0.044	0.79	***	10	6	0.60	0.205	0.50	**	10	6	0.60	0.205	0.50	**
13	Dependence in social contacts	10	8	0.80	0.044	0.79	****	10	8	0.80	0.044	0.79	***	10	7	0.70	0.117	0.66	***	10	7	0.70	0.117	0.66	***
14	Resources in social contacts	10	8	0.80	0.044	0.79	****	10	8	0.80	0.044	0.79	****	10	6	0.60	0.205	0.50	**	10	6	0.60	0.205	0.50	**
15	Other dependencies	10	6	0.60	0.205	0.50	**	10	7	0.70	0.117	0.66	***	10	5	0.50	0.246	0.34	*	10	5	0.50	0.246	0.34	*
16	Other resources	9	5	0.56	0.246	0.41	**	9	7	0.78	0.070	0.76	****	9	5	0.56	0.246	0.41	**	9	5	0.56	0.246	0.41	**
17	Burden through dependencies	10	9	0.90	0.010	0.90	****	9	7	0.78	0.070	0.76	****	9	6	0.67	0.164	0.60	***	9	6	0.67	0.164	0.60	***
		S-CVI/AVE 0.81						S-CVI/AVE 0.85			****			S-CVI/AVE 0.72						S-CVI/AVE 0.72					
		S-CVI/UA 0.12						S-CVI/UA 0.24						S-CVI/UA 0.00						S-CVI/UA 0.00					

^a^ I-CVI (content validity index) = number of experts providing a rating of 3 or 4/number of experts

^b^ p_c_ (probability of chance occurrence) = [N!/A!(N-A)!] × 0.5^N,^ N = number of experts; A = number of experts agreeing on a rating of 3 or 4

^c^ k* (modified kappa) = (I-CVI-p_c_)(1-p_c_)

^d^ Evaluation criteria for level of content validity: relationship between I-CVI and k*; excellent validity = I-CVI ≥ 0.78 and k* > 0.74 (****); good validity I-CVI < 0.78 and ≥ 0.60 and k* ≤ 0.74 (***); fair validity I-CVI < 0.6 and ≥ 0.40 and k* ≤0.59 (**); poor validity I-CVI < 0.4 and k* <0.40 (*)

**Table 9:** Content validity of the dimension “Physical discomfort”

		Relevance of the questions						Clarity of the questions						Meaningfulness of the answers						Completeness of the answers					
No	Content of the items	Number of experts	Number of ratings of 3 or 4	I-CVI^a^	p_b_	k* ^c^	Evaluation^d^	Number of experts	Number of ratings of 3 or 4	I-CVI^a^	p_c_^b^	k* ^c^	Evaluation^d^	Number of experts	Number of ratings of 3 or 4	I-CVI^a^	p_c_^b^	k* ^c^	Evaluation^d^	Number of experts	Number of ratings of 3 or 4	I-CVI^a^	p_c_^b^	k* ^***c***^	Evaluation^d^
1	Health problems generic	9	8	0.89	0.018	0.89	****	9	7	0.78	0.070	0.76	****	-	-	-	-	-	-	-	-	-	-	-	-
2	Pain	10	10	1.00	0.001	1.00	****	10	10	1.00	0.001	1.00	****	9	8	0.89	0.018	0.89	****	9	6	0.67	0.164	0.60	***
3	Depression	10	10	1.00	0.001	1.00	****	8	8	1.00	0.004	1.00	****	8	7	0.88	0.031	0.87	****	9	7	0.78	0.070	0.76	****
4	Hallucination/delusion	10	10	1.00	0.001	1.00	****	10	9	0.90	0.010	0.90	****	10	9	0.90	0.010	0.90	****	10	8	0.80	0.044	0.79	****
5	Sleep	10	10	1.00	0.001	1.00	****	10	8	0.80	0.044	0.79	****	10	8	0.80	0.044	0.79	****	10	7	0.70	0.117	0.66	***
6	Bowel elimination	10	10	1.00	0.001	1.00	****	10	9	0.90	0.010	0.90	****	10	8	0.80	0.044	0.79	****	10	8	0.80	0.044	0.79	****
7	Urinary elimination	10	10	1.00	0.001	1.00	****	10	8	0.80	0.044	0.79	****	10	9	0.90	0.010	0.90	****	10	8	0.80	0.044	0.79	****
8	Thirst/hunger	8	8	1.00	0.004	1.00	****	10	8	0.80	0.044	0.79	****	10	8	0.80	0.044	0.79	****	10	8	0.80	0.044	0.79	****
9	Need for movement	10	7	0.70	0.117	0.66	***	10	8	0.80	0.044	0.79	****	10	8	0.80	0.044	0.79	****	10	8	0.80	0.044	0.79	****
10	Breathing/circulatory problems	10	9	0.90	0.010	0.90	****	9	7	0.78	0.070	0.76	****	10	8	0.80	0.044	0.79	****	10	8	0.80	0.044	0.79	****
11	Discomfort	10	6	0.60	0.205	0.50	**	10	5	0.50	0.246	0.34	*	9	6	0.67	0.164	0.60	***	9	6	0.67	0.164	0.60	***
12	Other relevant health problems	9	8	0.89	0.018	0.89	****	8	6	0.75	0.109	0.72	***	8	5	0.63	0.219	0.52	**	8	6	0.75	0.109	0.72	***
13	Side effects of medication	9	9	1.00	0.002	1.00	****	9	6	0.67	0.164	0.60	***	9	9	1.00	0.002	1.00	****	9	8	0.89	0.018	0.89	****
		S-CVI/AVE 0.92						S-CVI/AVE 0.81			****			S-CVI/AVE 0.82						S-CVI/AVE 0.77					
		S-CVI/UA 0.62						S-CVI/UA 0.15						S-CVI/UA 0.08						S-CVI/UA 0.00					

^a^ I-CVI (content validity index) = number of experts providing a rating of 3 or 4/number of experts

^b^ p_c_ (probability of chance occurrence) = [N!/A!(N-A)!] × 0.5^N,^ N = number of experts; A = number of experts agreeing on a rating of 3 or 4

^c^ k* (modified kappa) = (I-CVI-p_c_)(1-p_c_)

^d^ Evaluation criteria for level of content validity: relationship between I-CVI and k*; excellent validity = I-CVI ≥ 0.78 and k* >0.74 (****); good validity I-CVI < 0.78 and ≥ 0.60 and k* ≤0.74 (***); fair validity I-CVI < 0.6 and ≥ 0.40 and  k* ≤ 0.59 (**); poor validity I-CVI < 0.4 and k* <0.40 (*)

**Table 10:** Content validity of the dimension “Communication”

		Relevance of the questions						Clarity of the questions						Meaningfulness of the answers						Completeness of the answers					
No	Content of the items	Number of experts	Number of ratings of 3 or 4	I-CVI^a^	p_c_^b^	k* ^c^	Evaluation^d^	Number of experts	Number of ratings of 3 or 4	I-CVI^a^	p_c_^b^	k* ^c^	Evaluation^d^	Number of experts	Number of ratings of 3 or 4	I-CVI^a^	p_c_^b^	k* ^c^	Evaluation^d^	Number of experts	Number of ratings of 3 or 4	I-CVI^a^	p_c_^b^	k* ^c^	Evaluation^d^
1	To express oneself verbally	10	10	1.00	0.001	1.00	****	10	10	1.00	0.001	1.00	****	10	8	0.80	0.044	0.79	****	10	8	0.80	0.044	0.79	****
2	To understand verbal communication	10	9	0.90	0.010	0.90	****	10	7	0.70	0.117	0.66	***	10	7	0.70	0.117	0.66	***	10	7	0.70	0.117	0.66	***
3	To understand written language	10	7	0.70	0.117	0.66	***	9	7	0.78	0.070	0.76	****	9	6	0.67	0.164	0.60	***	9	7	0.78	0.070	0.76	****
4	Hearing	10	10	1.00	0.001	1.00	****	10	10	1.00	0.001	1.00	****	10	9	0.90	0.010	0.90	****	10	9	0.90	0.010	0.90	****
5	Vision	10	10	1.00	0.001	1.00	****	10	10	1.00	0.001	1.00	****	10	8	0.80	0.044	0.79	****	10	9	0.90	0.010	0.90	****
6	Communication of wishes and needs	10	10	1.00	0.001	1.00	****	10	10	1.00	0.001	1.00	****	10	10	1.00	0.001	1.00	****	9	8	0.89	0.018	0.89	****
		S-CVI/AVE 0.93						S-CVI/AVE 0.91						S-CVI/AVE 0.81						S-CVI/AVE 0.83					
		S-CVI/UA 0.67						S-CVI/UA 0.67						S-CVI/UA 0.16						S-CVI/UA 0.00					

^a^ I-CVI (content validity index) = number of experts providing a rating of 3 or 4/number of experts

^b^ p_c_ (probability of chance occurrence) = [N!/A!(N-A)!] × 0.5^N,^ N = number of experts; A = number of experts agreeing on a rating of 3 or 4

^c^ k* (modified kappa) = (I-CVI-p_c_)(1-p_c_)

^d^ Evaluation criteria for the level of content validity: relationship between I-CVI and k*; excellent validity = I-CVI ≥ 0.78 and k* >0.74 (****); good validity I-CVI < 0.78 and ≥ 0.60 and k* ≤0.74 (***); fair validity I-CVI < 0.6 and ≥ 0.40 and k* ≤0.59 (**); poor validity I-CVI < 0.4 and k* <0.40 (*)

**Table 11:** Content validity of the dimension “Personality & life style before the onset of dementia”

		Relevance of the questions						Clarity of the questions						Meaningfulness of the answers						Completeness of the answers					
No	Content of the items	Number of experts	Number of ratings of 3 or 4	I-CVI^a^	p_c_^b^	k* ^c^	Evaluation^d^	Number of experts	Number of ratings of 3 or 4	I-CVI^a^	p_c_^b^	k* ^c^	Evaluation^d^	Number of experts	Number of ratings of 3 or 4	I-CVI^a^	p_c_^b^	k* ^c^	Evaluation^d^	Number of experts	Number of ratings of 3 or 4	I-CVI^a^	p_c_^b^	k* ^c^	Evaluation^d^
1	Personality prior to the onset of dementia	10	9	0.90	0.010	0.90	****	9	8	0.89	0.018	0.89	****	10	8	0.80	0.044	0.79	****	10	7	0.70	0.117	0.66	***
2	Stress and frustration tolerance	10	9	0.90	0.010	0.90	****	10	8	0.80	0.044	0.79	****	10	9	0.90	0.010	0.90	****	10	8	0.80	0.044	0.79	****
3	Coping with stressful situations before the onset of dementia	10	10	1.00	0.001	1.00	****	10	8	0.80	0.044	0.79	****	10	9	0.90	0.010	0.90	****	10	9	0.90	0.010	0.90	****
4	Past situations or events related to negative emotions	10	9	0.90	0.010	0.90	****	10	9	0.90	0.010	0.90	****	10	9	0.90	0.010	0.90	****	10	10	1.00	0.001	1.00	****
5	Past situations or events related to positive emotions	9	9	1.00	0.002	1.00	****	9	9	1.00	0.002	1.00	****	9	7	0.78	0.070	0.76	****	9	9	1.00	0.002	1.00	****
6	Leisure activities preferred by the resident prior to dementia	9	8	0.89	0.018	0.89	****	9	9	1.00	0.002	1.00	****	9	8	0.89	0.018	0.89	****	9	8	0.89	0.018	0.89	****
7	Type of job/housework before dementia	9	9	1.00	0.002	1.00	****	9	9	1.00	0.002	1.00	****	9	7	0.78	0.070	0.76	****	9	8	0.89	0.018	0.89	****
8	Important routine rhythms or established daily rituals	9	9	1.00	0.002	1.00	****	9	9	1.00	0.002	1.00	****	9	9	1.00	0.002	1.00	****	8	8	1.00	0.004	1.00	****
		S-CVI/AVE 0.95						S-CVI/AVE 0.92						S-CVI/AVE 0.87						S-CVI/AVE 0.90					
		S-CVI/UA 0.50						S-CVI/UA 0.50						S-CVI/UA 0.13						S-CVI/UA 0.38					

^a^ I-CVI (content validity index) = number of experts providing a rating of 3 or 4/number of experts

^b^ p_c_ (probability of chance occurrence) = [N!/A!(N-A)!] × 0.5^N,^ N = number of experts; A = number of experts agreeing on a rating of 3 or 4

^c^ k* (modified kappa) = (I-CVI-p_c_)(1-p_c_)

^d^ Evaluation criteria for level of content validity: relationship between I-CVI and k*; excellent validity = I-CVI ≥ 0.78 and k* >0.74 (****); good validity I-CVI < 0.78 and ≥ 0.60 and k* ≤0.74 (***); fair validity I-CVI < 0.5 and ≥ 0.40 and k* ≤0.59 (**); poor validity I-CVI < 0.4 and k* <0.40 (*)

**Table 12:** Content validity of the dimension “Mood & emotions”

		Relevance of the questions						Clarity of the questions						Meaningfulness of the answers						Completeness of the answers					
No	Content of the items	Number of experts	Number of ratings of 3 or 4	I-CVI^a^	p_c_^b^	k* ^c^	Evaluation^d^	Number of experts	Number of ratings of 3 or 4	I-CVI^a^	p_c_^b^	k* ^c^	Evaluation^ad^	Number of experts	Number of ratings of 3 or 4	I-CVI^a^	p_c_^b^	k* ^c^	Evaluation^d^	Number of experts	Number of ratings of 3 or 4	I-CVI^a^	p_c_^b^	k* ^c^	Evaluation^d^
1	Boredom	10	10	1.00	0.001	1.00	****	10	6	0.60	0.205	0.50	**	10	8	0.80	0.044	0.79	****	10	9	0.90	0.010	0.90	****
2	Anxiety	10	10	1.00	0.001	1.00	****	10	6	0.60	0.205	0.50	**	10	8	0.80	0.044	0.79	****	10	9	0.90	0.010	0.90	****
3	Exhaustion	10	9	0.90	0.010	0.90	****	10	7	0.70	0.117	0.66	***	10	8	0.80	0.044	0.79	****	10	9	0.90	0.010	0.90	****
4	Impression of loneliness or isolation	10	9	0.90	0.010	0.90	****	10	6	0.60	0.205	0.50	**	10	7	0.70	0.117	0.66	***	10	9	0.90	0.010	0.90	****
5	Trusting relationships	10	7	0.70	0.117	0.66	***	10	5	0.50	0.246	0.34	*	10	8	0.80	0.044	0.79	****	10	6	0.60	0.205	0.50	**
6	Amount of occupation	10	8	0.80	0.044	0.79	****	9	7	0.78	0.070	0.76	****	10	8	0.80	0.044	0.79	****	10	5	0.50	0.246	0.34	*
7	Type of occupation	10	8	0.80	0.044	0.79	****	10	7	0.70	0.117	0.66	***	10	9	0.90	0.010	0.90	****	10	9	0.90	0.010	0.90	****
		S-CVI/AVE 0.87						S-CVI/AVE 0.64						S-CVI/AVE 0.80						S-CVI/AVE 0.80					
		S-CVI/UA 0.29						S-CVI/UA 0.50						S-CVI/UA 0.00						S-CVI/UA 0.00					

^a^ I-CVI (content validity index) = number of experts providing a rating of 3 or 4/number of experts

^b^ p_c_ (probability of chance occurrence) = [N!/A!(N-A)!] × 0.5^N,^ N = number of experts; A = number of experts agreeing on a rating of 3 or 4

^c^ k* (modified kappa) = (I-CVI-p_c_)(1-p_c_)

^d^ Evaluation criteria for level of content validity: relationship between I-CVI and k*; excellent validity = I-CVI ≥ 0.78 and k* >0.74 (****); good validity I-CVI < 0.78 and ≥ 0.60 and k* ≤0.74 (***); fair validity I-CVI < 0.6 and ≥ 0.40 and k* ≤0.59 (**); poor validity I-CVI < 0.4 and k* <0.40 (*)

**Table 13:** Content validity of the dimension “Environmental influences”

		Relevance of the questions						Clarity of the questions						Meaningfulness of the answers						Completeness of the answers					
No	Content of the items	Number of experts	Number of ratings of 3 or 4	I-CVI^a^	p_c_^b^	k* ^c^	Evaluation^d^	Number of experts	Number of ratings of 3 or 4	I-CVI^a^	p_c_^b^	k* ^c^	Evaluation^d^	Number of experts	Number of ratings of 3 or 4	I-CVI^a^	p_c_^b^	k* ^c^	Evaluation^d^	Number of experts	Number of ratings of 3 or 4	I-CVI^a^	p_c_^b^	k* ^c^	Evaluation^d^
1	Surroundings	9	8	0.89	0.018	0.89	****	8	5	0.63	0.219	0.52	**	-	-	-	-	-							
2	Lighting	10	9	0.90	0.010	0.90	****	10	7	0.70	0.117	0.66	***	10	8	0.80	0.044	0.79	****	10	5	0.50	0.246	0.34	*
3	Noises	10	9	0.90	0.010	0.90	****	10	8	0.80	0.044	0.79	****	10	8	0.80	0.044	0.79	****	10	7	0.70	0.117	0.66	***
4	Smells	10	7	0.70	0.117	0.66	***	10	8	0.80	0.044	0.79	****	9	6	0.67	0.164	0.60	***	9	7	0.78	0.070	0.76	****
5	Stimuli	10	8	0.80	0.044	0.79	****	10	7	0.70	0.117	0.66	***	7	5	0.71	0.164	0.66	***	10	8	0.80	0.044	0.79	****
6	Atmosphere	10	6	0.60	0.205	0.50	**	9	6	0.67	0.164	0.60	***	10	7	0.70	0.117	0.66	***	10	8	0.80	0.044	0.79	****
7	Security	10	9	0.90	0.010	0.90	****	10	9	0.90	0.010	0.90	****	9	7	0.78	0.070	0.76	****	10	7	0.70	0.117	0.66	***
8	Private space	10	9	0.90	0.010	0.90	****	10	9	0.90	0.010	0.90	****	10	9	0.90	0.010	0.90	****	10	7	0.70	0.117	0.66	***
9	Opportunities for contact	10	9	0.90	0.010	0.90	****	10	9	0.90	0.010	0.90	****	10	9	0.90	0.010	0.90	****	10	7	0.70	0.117	0.66	***
10	Significant carer/caregivers	9	9	1.00	0.002	1.00	****	9	9	1.00	0.002	1.00	****	9	9	1.00	0.002	1.00	****	9	9	1.00	0.002	1.00	****
11	Continuity of being in contact with significant carer	8	7	0.88	0.031	0.87	****	8	7	0.88	0.031	0.87		9	7	0.78	0.070	0.76	****	9	8	0.89	0.018	0.89	****
		S-CVI/AVE 0.85						S-CVI/AVE 0.81						S-CVI/AVE 0.80						S-CVI/AVE 0.76					
		S-CVI/UA 0.09						S-CVI/UA 0.09						S-CVI/UA 0.10						S-CVI/UA 0.10					

^a^ I-CVI (content validity index) = number of experts providing a rating of 3 or 4/number of experts

^b^ p_c_ (probability of chance occurrence) = [N!/A!(N-A)!] × 0.5^N,^ N = number of experts; A = number of experts agreeing on a rating of 3 or 4

^c^ k* (modified kappa) = (I-CVI-p_c_)(1-p_c_)

^d^ Evaluation criteria for level of content validity: relationship between I-CVI and k*; excellent validity = I-CVI ≥ 0.78 and k* >0.74 (****); good validity I-CVI < 0.78 and ≥ 0.60 and k* ≤0.74 (***); fair validity I-CVI < 0.6 and ≥ 0.40 and k* ≤0.59 (**); poor validity I-CVI < 0.4 and k* <0.40 (*)

For the challenging behaviour dimension, all 18 questions showed good validity regarding relevance and 16 regarding clarity. One item was considered fair and one poor in terms of clarity. For the answers, 16 of 18 items showed excellent validity in terms of meaningfulness and 14 in terms of completeness. Two answers were considered good regarding meaningfulness and 3 regarding completeness. One answer item showed fair validity in terms of completeness. Altogether, 5 complete items (question plus answer options, no. 12 and no. 17) needed modification (Table 5).

Six of 7 questions for the dimension *cognitive status* were considered excellent (relevance and clarity). Two items had good validity (no. 4 for relevance; no. 4a for clarity). One answer (no. 3) showed fair values for meaningfulness, and two items were considered good in terms of completeness (no. 1 and no. 4a). In total, 6 complete items (questions and answers) needed some modification (Table 6).

All questions and all answers for the *physical health status* dimension were considered relevant or meaningful (excellent validity). All questions but one showed excellent validity in terms of clarity (no. 5a showed good validity). Three of 4 answers were considered excellent in terms of completeness, and one showed good validity (no. 5a). Overall, only one complete item (5a) needed revising (Table 7).

Eleven of 13 questions for the dimension *physical discomfort* were considered excellent, one good and one fair regarding relevance. Ten questions were rated excellent, 2 good and one poor in terms of clarity. Ten of 12 answers were considered excellent, one good and one fair regarding meaningfulness, and 8 were rated excellent and 2 good for completeness. Altogether, 6 complete items needed modification (Table 8).

For the dimension *independence in everyday life,* 12 of the 17 questions were rated excellent regarding relevance, 3 good and 2 fair. Fifteen questions showed excellent validity in terms of clarity, one showed good validity, and one had fair validity. Only 7 answers were considered excellent in terms of meaningfulness, whereas 5 were good, 5 fair and one poor. Six answers were rated excellent for completeness, 10 good, and one fair. Overall, 14 of the 17 complete items needed some degree of modification (Table 9).

Five of 6 questions for the dimension *communication* were considered to have excellent validity in terms of relevance and clarity. One question showed good validity for relevance and one for clarity. Four answers had excellent ratings and two had good ratings for meaningfulness and completeness. In total, 2 complete items needed modification (Table 10).

For the dimension *personality & lifestyle before the onset of dementia ,* all questions were considered relevant and clear (excellent validity). Additionally, all answers showed excellent values in terms of meaningfulness, and all but one in terms of completeness. One answer showed good validity and needed modification (Table 11).

The relevance of 6 of the 7 questions in the *mood & emotions* dimension were rated excellent and one good. In contrast, only one question showed excellent validity, two indicated good validity, two were fair and one poor in terms of clarity. For meaningfulness and completeness, 6 answers were rated excellent for both. One answer showed good validity for meaningfulness. One answer showed fair validity and one poor validity for completeness. Altogether, all of the items needed modification to different degrees (Table 12).

For the *environmental influences* dimension*,* 9 of 10 questions showed excellent, two good and one fair validity regarding relevance. Seven questions had excellent, two good and one fair validity values in terms of clarity. For meaningfulness, 7 answers were considered excellent and 3 good. For completeness, 5 answers were considered excellent, 4 good and 1 poor. Overall, 9 complete items had to be revised (Table 13).

The modifications to the instrument were made based on the CVI scores and the free text comments of the expert panel (Table 3). The changes were mainly related to a reduction in the questions and a specification and clarification of the answers. The items in part 1 that focused on the experiences and reactions of the social environment regarding challenging behaviour were the most criticized. These criticisms resulted in the rejection of two items and a merging of other items. In part 2 of the instrument (capturing the triggers of the behaviour), the subject area *independence in everyday life* was fundamentally revised. The main criticism was that the items were too rich in detail about care dependency and that the relation of the dimension to challenging behaviour was not clear. The experts recommended replacing the assessment of dependency with an assessment of the consequences or experiences of dependency resulting in the development of challenging behaviour. These consequences included the “stress/burden of the person with dementia due to being limited in self-care ability” and “stress/burden related to the care activities themselves”. Additionally, the subject area *mood* & *emotions* was further refined. The experts mentioned several times in the free text that because of the large number of questions, it seemed difficult to maintain an overview of all potentially important factors related to the development of challenging behaviour. Similarly, the objective of the assessment seemed to fade during the assessment process, and the reference to the challenging behaviour decreased. To maintain focus on the aim of the instrument during the assessment process and to illustrate the relationship between the items and challenging behaviour, overarching key questions were developed for all subject areas. These key questions introduced revised assessment dimensions (Table 14). The modified version of the IdA® (version 2.0) included 72 items and was presented for discussion during the subsequent workshop with the health care practitioners.

**Table 14:** Content of the IdA® and the guiding questions (Version 3)

Dimension	Guiding questions	Content of dimension
*Part 1 Description of the behaviour*		
Assessment of challenging behaviour	Which type of challenging behaviour is observed?	Naming and description of the behaviour; description of the circumstances under which the behaviour occurs; quantification of behaviour; denomination of the consequences of the behaviour
*Part 2 Capturing the triggers of the behaviour*		
Cognitive status	Could the challenging behaviour be explained by the type or stage of dementia? Could the identified cognitive impairments explain the challenging behaviour?	Events from the past, information about oneself, present living situation, sense of time, orientation in important rooms, complete activities, recognition of important everyday items, recall of information received a day or less before, recognition of important everyday items
Physical health status & discomfort	Could the identified physical impairment or discomfort somehow be related to the challenging behaviour?	Mobility, fluid and food intake, excretion functions, sleep, vital physical functions, depression, pain, delusions/hallucinations, medications with adverse side effects
Independence in everyday life	Could the identified stressful/burdening dependencies in everyday life activities have provoked the challenging behaviour?	Emotional burden/stress of care dependencies and care interventions
Communication	Could the identified comprehension/communication difficulties have triggered/provoked the challenging behaviour? Is it possible that the behaviour itself presents a form of communication and to explain the behaviour accordingly?	Hearing/seeing well, language of communication, comprehensibility of speech, quality of verbal communication, understanding of verbal/written communication, contact with others, communication of personal wishes/desires
Personality & lifestyle before the onset of dementia	Could the challenging behaviour be an expression of the resident’s personality? Could the challenging behaviour be related to past life events or the person’s former lifestyle? Could the challenging behaviour be a reaction to stress?	Personality before the onset of dementia, stress tolerance, frustration tolerance, management of stressful situations before dementia; Events connected with negative emotions or threatening events, events associated with positive impact/emotions, leisure time before the onset of dementia, occupation, daily rhythm/daily rituals with unique importance
Mood & emotions	Could the challenging behaviour be an expression of certain moods and emotions? Could the challenging behaviour serve as self-stimulation?	Fear, tiredness/exhaustion, closer relationship to the resident, relationship showing confidence, safety, times without occupation, boredom, occupational activities/leisure time activities/structure of the day not matching the residents´ personal preferences
Environmental influences	Could the challenging behaviour be related to certain environmental characteristics? Could the challenging behaviour be related to a lack of sense of security and familiarity? Could staff structure have an impact on the challenging behaviour?	Lighting, noise in the surroundings, smells, furnishing, privacy, contact with others, positive stimuli/stimulations, preference of caregiver, continuity of primary caregivers

### 2nd evaluation: Workshop with health care workers

#### Sample

Seventeen persons from 10 nursing homes participated in the workshop. Most of them were nurses, and a few had a geriatric background, were leaders of the unit or nursing home, or were quality managers. No further information about the participants was collected.

#### Results

The discussion with the health care workers was very helpful for determining how the items were understood and interpreted. The language was simplified based on the results of the workshop. The subject areas “communication” and “independence in everyday life” again required further clarification. Some items were perceived to be too broad; the participants expressed a need for more differentiated and detailed information. Difficulty summarizing the large amount of information obtained was the strongest piece of criticism. Two main points were discussed. First, the requirements needed to answer the items required further consideration. Some items could only be answered with more in-depth observations of the person with dementia, e.g., their cognitive abilities, or further information was needed from family members or doctors. For example, the domain “Personality & lifestyle before the onset of dementia” contained questions that could not be answered without knowing the person well and for a long period of time. The nursing home care staff was often dependent on information provided by family members or significant others, if present. In some cases, these informants were not available, and the necessary information to answer the IdA® questions could not be obtained. Other items led to actions such as speaking with the occupational therapist about activities or a change in care routines. Second, the participants wanted to have an overview of all relevant aspects that could be associated with the challenging behaviour in the particular individual. To address the first criticism, each item received a new assessment criterion called “what has to be done?”. This question included three selection options: a) further clarification needed, b) action needed to plan, and c) item remains important. Referring to the second point of criticism, key questions with the corresponding significant content were presented on a separate page through bullet points. These modifications resulted in version 3.0 of the IdA®, which contained 7 domains with 89 items and three parts.

### Stage III evaluation

#### Sample

Sixty of the 229 questionnaires were returned. One ward unit withdrew from the study because of being transferred from one location to another. The response rate varied considerably between nursing homes (7% to 58%). The reasons for non-response included holiday time, illness, and a lack of time. The respondents’ characteristics are shown in Table 15. The characteristics of the sample reflect the population of the care staff in German nursing homes.

**Table 15:** Characteristics of the sample in the evaluation study

Characteristics of the sample *n* = 60	
Women, n [%] *n* = 56	50 [89]
Age, n [%], years, *n* = 51, 8 missing	
Up to 19	1 [2]
20–24	5 [10]
25–29	8 [16]
30–34	3 [6]
35–39	1 [1]
40–44	10 [20]
45–49	8 [16]
50–54	9 [17]
55–59	3 [6]
60 and over	3 [6]
Profession n [%], *n* = 55, 5 missing	
Registered nurses	23 [42]
Nursing aides	22 [40]
Other	10 [18]
Working hours per week, x [SD], *n* = 53, 7 missing	28.8 [9.3]

## Results

### Structure of the IdA®

The majority of the respondents positively rated 9 of the 13 structural aspects of the IdA®. Three aspects were rated positively by 40% to 50% of the respondents. However, 71% of the respondents rated the time aspect negatively. The IdA® did not have to be applied by registered nurses only (67%), but the interpretation of information from the IdA® required broad knowledge of dementia (Table 16).

**Table 16:** Evaluation of the structural aspects of the IdA®

Structural aspects	Items	Agreement	n
Clarity	Very clear	68%	57
	Meaningfully constructed	70%	57
	Not too inconvenient	66%	56
	Allows prompt access to resident information	61%	56
Meaningfulness	Contains only information relevant to care	42%	57
	No double documentation needed	49%	56
Completeness	Complete	81%	57
Length/scope	Not too detailed	53%	57
	Not time consuming	29%	57
	Not too extensive	42%	57
Comprehensibility	Clear language	75%	56
	Does not require comprehensive training	53%	58
	Not too complicated	69%	57
Requirements	Requires knowledge of dementia	60%	58
	Completed only by registered nursing staff	33%	58

The detailed ratings of each domain of the IdA® showed that the majority of the respondents provided mostly positive assessments of the completeness (71–80%) of the information, the relevance of the information for daily care (64–76%) and the contribution of the IdA® domains for internal (71–82%) and external communication (59–67%).

### Benefits of the IdA® for daily care

Six aspects, asked with 8 items, pertained to the benefit of the IdA® for the daily care of residents with dementia. The majority of the respondents (min. 50%) rated six items positively. However, most of the respondents perceived the IdA® as not capable of indicating quality of care and as less supportive of daily communication with other health care workers (Table 17).

**Table 17:** Evaluation of the benefit of the IdA® for daily care of residents with dementia and challenging behaviour

Aspects of daily care	Items	Agreement	n
Time	Does not reduce valuable time for care	53%	58
Information aid	Provides information about residents’ bio-psycho-social information	80%	56
	The IdA® can be read by others	55%	58
Communication aid	Is helpful for communication with colleagues	60%	58
	Is helpful for communication with other health care workers	46%	57
Planning aid	Supports planning of care	64%	58
Evaluation aid	Indicates good care	36%	58
Memory aid	Is a good memory aid	67%	58

The overall rating of the IdA® was positive. Two of three respondents (64%) rated the IdA® as good to very good. When asked “Would you continue using the assessment tool after the testing phase?”, 28 of 47 (56%) reported that they would use the IdA®, whereas 19 said they would not. If the IdA® continued to be used together in a team, 84% of the users said that they would keep using the tool.

### The IdA® as a tool for decision making

For decision making regarding the causes of challenging behaviour, it was important for the care staff to have individualized information about the residents. In total, 74% of the respondents confirmed the statement *“The IdA® gives me the opportunity to express the individuality of the resident with dementia”*. The IdA® seemed to mostly stimulate reflection on the residents’ behaviour. The IdA® included aspects related to behaviour that the majority of the respondents had not considered before; furthermore, it helped describe the residents’ behaviour to others and to provide another perspective on the behaviour. Two-thirds of the respondents thought that the IdA® was relevant to their daily work. Regarding the statements of whether the IdA® had changed their own behaviour, helped plan interventions, provided unknown information or improved understanding of the behaviour, between 44% and 47% of the respondents agreed with the statements. Unfortunately, the respondents did not experience an improvement in the behaviour of the residents (Table 18).

**Table 18:** Benefits of the IdA® for decision making

Benefit for decision making	Agreement	n =
The IdA® has encouraged me to think more intensively about the residents’ behaviour	71%	59
The IdA® contains factors that I had never thought about in the context of residents’ problem behaviour	66%	59
I see a relationship between the IdA® and my daily work	64%	58
By using the IdA®, I see the behaviour of the residents with dementia from another perspective	63%	59
With the IdA®, I can better describe the residents’ problem behaviour to others	58%	59
Planning interventions to address the residents´ behaviour is easier with the information provided by the IdA®	47%	58
My behaviour towards the residents has changed since the introduction of the IdA®	47%	58
The IdA® provides information about the residents that I did not know before	45%	58
Since the introduction of the IdA®, I have a better understanding of the behaviour of the residents with dementia	44%	57
I feel that the behaviour of the residents has changed positively since the introduction of the IdA®	26%	58
The IdA® gives me the opportunity to express the individuality of the resident with dementia	74%	58

## Discussion

The purpose of this study was to develop a nursing assessment of the triggers and causes of challenging behaviour among residents with dementia as a diagnostic guideline and to assess its content validity, feasibility and practicability. We used the approach suggested by Lynn [26], which is broadly applied in health care and nursing research [47–49]. Although Beckstead [50] questioned the meaning of content validity for establishing the validity of an instrument, the two-stage approach is widely accepted in the methodological literature and is considered a requirement for the development of new instruments [31, 51–53]. The main criticism refers to the calculation of the agreement indices and the significance of the number of experts for the risk of error. We used the modified kappa as an index that adjusts for chance agreement. This index provides information about the degree of agreement beyond chance. Questions concerning whether the use of other approaches, such as multirater kappa, would result in more correct agreement cannot be answered here. However, the agreement indices are only one part of the process of assessing content validity and should not be the only reason for the rejection or modification of items. The comments of the experts helped form judgements about the type of problems that existed for specific items. Thus, CVI together with modified kappa and written comments of the experts added rigour to the validation process of the content of IdA®.

Content validity expresses the representation of current available knowledge in the construct of interest [28]. Together with face validity, content validity represents the minimum quality requirement for an instrument. However, despite its limited value within the validity hierarchy [50, 54], content validity is an important quality indicator of an instrument’s validity and provides insight into its feasibility and practicability [32, 51, 54]. In this study, the development process of the instrument and the evaluation of its content validity supported the validity of the IdA® and provided a strong foundation from which to begin further examination of its validity and reliability.

To assess the content validity of the IdA®, it was important to assess different attributes of both the questions and the answer options. The experts perceived the majority of the questions as relevant; however, more weaknesses with the answer options were identified. The experts’ judgements together with the comments provided detailed information about the strengths and weaknesses of each item and led to well-reasoned modifications.

The decision to assess more than only the relevance of an item was shown to be very helpful in the development process. The experts distinguished between the relevance of the content (particular trigger or cause of behaviour) and the clarity of the wording. Most of the questions were considered relevant, but the phrasing was criticized. This differentiation facilitated the modifications and improved their comprehensibility. The responses received more negative criticisms than the questions. For example, the relevance of the item *asking about pain* was confirmed, and the answer options were meaningful. However, in the experts´ opinion, some important aspects relevant to pain assessment were not considered. Additionally, in some cases, the written comments indicated that there were problems with comprehension – the intended content was somewhat misunderstood. This information led to a more precise phrasing of the item.

The importance of the competency of the expert is crucial. Very different factors can be used to confirm a person as an expert, and there are no rules on how to define an expert. Usually, an expert is defined as a person who represents the content of interest in science or practice. In the context of assessment instruments, knowledge about the methodology of assessment is very helpful, and input from stakeholders from additional application fields can be useful [52]. In the first expert panel of this study, we focused on experiences and knowledge in dementia care, in nursing care in the context of nursing homes and in using or developing assessment instruments. The strong focus on nursing was requested, as we expected that those experts would help to develop an instrument with content that is meaningful, understandable and practicable for nurses and the nursing field. We could not clarify the extent to which the narrow focus on nursing field influenced the rating of relevance of the IdA® items and dimensions in favour of more physical and ADL topic than psycho-social aspects. We assume a small risk of bias since most the experts had additional qualifications and professions beyond nursing.

The disadvantage of the development process was the time and effort needed to assess the substantial number of items, as the process could not be replicated after the modifications suggested by the first expert panel had been made. Because the second review process occurred through a workshop with health care workers, individual ratings of the assessment items were not feasible. Thus, the effect of modifying the IdA® items on the CVI obtained in the first assessment could not be quantified, which is a limitation of the study.

The health care practitioner’s comments and discussion contributed significantly to the item clarity and adaptability to nursing practice. In this second modification, aspects such as problems obtaining the information needed to answer the questions were strongly discussed. For example, the domain “Personality & lifestyle before the onset of dementia” contained questions that could not be answered without knowing the person well and for a long period of time. The nursing home care staff was often dependent on information provided by family members or significant others, if present. In some cases, these informants were not available, and the information needed to answer the IdA® questions could not be obtained. This is not a problem specific to IdA® but is a general problem of assessments of complex and multifaceted information. The creation of a professional environment in which the observation, collection, communication, reflection and appraisal of essential information is an obvious part of the nursing process can overcome this challenge. IdA® should not be the start of this process, but it supports this process for a specific topic (dementia or challenging behaviour) and in a specific phase of this process (mainly complementary information collection, communication, reflection and appraisal of information). Although the CVI could not be calculated in this second expert round, the advantage of this round was the clear practice perspective, the simplification of the wording and the willingness of the carers to collect, provide and share information.

The majority of the IdA® users reported that the tools were well structured, clear, complete and comprehensive. The content appeared to be ambiguous. For almost 60% of the users, the relevance of the IdA® information for dementia care seemed to be unclear. This finding was consistent with the evaluation that the IdA® required comprehensive knowledge about dementia and challenging behaviour. This feedback was important for the future implementation of the IdA®. The findings highlighted the insufficient knowledge of the staff working in nursing homes about the triggers of challenging behaviour. Particular emphasis should be placed on expressing the relationships between the IdA® variables and their relevance to the development of challenging behaviour. As previously found, the qualification of the staff caring for people with dementia is known to influence the effectiveness of dementia-specific approaches [55]. However, how the staff experiences challenging behaviour and how they place this behaviour in context also influence their understanding of causes and triggers. In their study, Dupuis et al. [56] showed a complex process of interpretation through which nurses use several filters. The lens of pathology and the influence of cognition on the construction of meaning of challenging behaviour are very dominant. Thus, it is important that nursing home staff are supported in developing more person-centred attitudes towards persons showing challenging behaviour. The combination of evidence-based knowledge about triggers, supported by IdA®, and the reinforcement of dementia-friendly attitudes are a promising basis for the best care for those people.

The IdA® was time consuming because it covered a wide range of information on potential causes, and these causes are multifactorial. The care staff needed time to answer the questions, gather any missing information, discuss potential hypotheses on the causes of behaviours, and then plan interventions. This process required a substantial amount of time at a point when nursing care is characterized by a lack of time [57]. This conflict could not be resolved, as the broadness of the themes and items was necessary to trace the triggers and causes of behaviour [58]. Changes in routine processes, changes in the prioritization of tasks and facilitation of a professional self-understanding that incorporates analytic thinking processes into nursing tasks could help address this conflict. An important form of preparation for the implementation of IdA® is to analyse current practices of nursing assessment and documentation. Most of the information needed for IdA® already exists. Nurses should ensure that the existing information is valid and can be easily linked with the questions on IdA®. This process has the potential to save time. We also expect that the frequent use of IdA® will result in better management of the instrument and better prioritization of the parts of IdA® relevant to a particular person with dementia. We analysed this aspect in a recently completed intervention study [21, 59].

The IdA® should be viewed as a tool that offers support and assistance for carers in understanding the challenging behaviour exhibited by persons with dementia. This tool provides guidelines for navigating a complex network of widely varying potential causes and triggers of these behaviours. It is important to mention that the IdA® does not provide exact solutions for the problem but rather helps generate a hypothesis (one or more) about the causes of challenging behaviour. This type of support is important in Germany, as nurses act practically independently in nursing homes. In Germany, a general practitioner (GP) spends an average of less than 2 h peer week in nursing homes, caring for 20 residents during this time period [60]. Because of the limited time and the high number of residents with challenging behaviours, contact with the GP is mainly restricted to acute or severe situations and focuses mainly on medical and pharmacological questions [61]. Bartholomeyczik et al. [15] found that nurses are only moderately satisfied with GP consultation times for residents with dementia, and only 13% of GPs wanted to participate in case conferences very often or often. In that study, only one GP participated and contributed to one case conference. Consultations with psychologists are unusual. This situation differs from the situation in the Netherlands, for example, where an interdisciplinary team is responsible for managing challenging behaviour [16], and in the UK, where the psychologist has a key role in this context [17].

The IdA® asks for a substantial amount of information about the person with dementia, and therefore different sources of information are important to obtaining a comprehensive understanding of the person, e.g., reports from different shift workers and carers, family and friends, therapists and persons with dementia themselves. Hypotheses regarding the causes or triggers of a behaviour are not formed as a result of a deduction of “hard facts” but rather a construction of plausible and preliminary presumptions. The effects of the action deduced from the hypotheses verify the accuracy of these estimates. Discussing the pros and cons of a hypothesis is therefore necessary to properly apply the IdA®. Favourable opportunities for these discussions include team meetings or case conferences [22]. The initial experiences in which the IdA® was used during case conferences in nursing homes showed that the care staff benefitted from the exchange of individual views and experiences. They were able to realize that residents showed different behaviours with different staff members, and this realization facilitated the recognition of helpful and unhelpful approaches to the interactions between residents and staff. Self-reflection was supported. Residents´ behaviour was no longer considered a personal attack but could be perceived from a broader context. The IdA® helped to more precisely describe the behaviour of residents with dementia as well as the circumstances of behaviour and supported communication with physicians. Through the IdA®, gaps in knowledge regarding the residents became apparent [55]. Thus, the IdA® has the potential to support carers’ self-efficacy and thus reduce their burden in managing challenging behaviour [18]. The effectiveness of using the IdA® within case conferences is currently being examined in a cluster randomized controlled trial in Germany [21, 59].

The IdA® is not a typical measurement instrument that can be easily tested using all routine psychometric properties (e.g., inter-rater reliability, criterion validity, structure analysis). The outcome of the IdA® is not a risk status or physical status that can be prospectively validated or compared to a gold standard. The results of the IdA® as a whole include the formulation of hypotheses about the relationship between the information obtained from the IdA® and challenging behaviour. The “truth” can be verified only if the derived conclusions (hypothesis) lead to the expected effects. The rejection or confirmation of the hypothesis also depends on the choice of the right intervention (e.g., ways to communicate or changes in medication or enjoyable activities) and on the correct execution of the intervention. This requires knowledge about effective interventions and skills and technique in delivering the interventions.

In addition, the effects in each person and situation could differ: challenging behaviour could be reduced or stabilized, the right interactions could be implemented when the behaviour occurs, the behaviour could be integrated into daily living in the nursing home unit, the burden could be relieved for persons in the patient’s environment, the quality of life of the person showing challenging behaviour could be maintained or the challenging behaviour could be better understood or re-interpreted. When the conclusion does not lead to the desired effects, the reasons can vary from incorrect hypotheses, incorrect interventions, or both; incorrect, missing or unimportant items on the IdA®; or a lack of availability of significant information. The evaluation of reliability also has some challenges. For IdA®, as a team instrument, the evaluation of inter-rater reliability is less meaningful, except for some specific aspects such as pain, cognitive status or some behavioural characteristics. A form of intergroup reliability could be an alternative, but the time needed for conducting repeated team measurements should be taken into account. Evaluation of test-retest for the formulated hypotheses could make more sense and help to overcome the previously discussed problems with the interpretation of validity.

### Limitations

The study has several limitations. Due to the many items included in the IdA®, it was not possible to conduct additional expert rounds, as suggested by Lynn [26], or to calculate CVIs after each evaluation round. Thus, whether the modifications led to improvements in the content validity remains unknown. Content validity is the weakest form of validity, and therefore this study is a first step in establishing the validity of the IdA®.

We used the CVI and modified kappa for the assessment of agreement, the merit of which has been criticized. Here, we do not know whether e.g. the multirater kappa statistic would provide more valid agreement indices. Therefore, different indices were used to decide the item modifications.

We included in the development process mainly professionals from the nursing field, since our priority was an instrument that covers information relevant to nursing care and can be managed by nurses. The involvement of other disciplines might influence the content and the content validity evaluation of IdA®.

The size of the care staff sample was small, and thus the interpretation of the results was limited.

Additionally, the delivery time was very short (6 weeks); this brief duration might have influenced the correct application of the IdA® and led to over- or underestimations of the IdA® quality. Conclusions regarding the experiences with the IdA® over time cannot be made.

## Conclusion

The IdA® is a nursing assessment tool that focuses on the triggers and causes of challenging behaviour. The comprehensive literature review, strong theoretical foundation, and the two evaluations representing specific content expertise as well as practical perspectives contribute to the content validity of the IdA®. The IdA® is recommended to be used as a team instrument, as the information collected with the IdA® requires observations from different caregivers, situations and time periods to create a comprehensive understanding of the persons exhibiting challenging behaviour.

## Additional files

Additional file 1: English version of IdA® (IdA-E®) (IdA_english_version.pdf). (PDF 2867 kb)

Additional file 2: Translation process of the German IdA ® into English (IdA-E®) using a forward and backward translation process. (PDF 516 kb)
