# Supplementary material for: Development and evaluation of the content validity, practicability and feasibility of the Innovative dementia-oriented Assessment system for challenging behaviour in residents with dementia
Source: BMC Health Serv Res. 2017 Aug 14;17:554. doi: 10.1186/s12913-017-2469-8 (PMC5557472; doi:10.1186/s12913-017-2469-8)
Supplement: Supplementary file 1 — English version of IdA® (IdA-E®) (IdA_english_version.pdf). (PDF 2867 kb) [file 12913_2017_2469_MOESM1_ESM.pdf]

1. *Which kind of challenging behaviour is observed?*

- ❑ **Passive behaviour** — for example to withdraw oneself, to be apathetic, not reacting, not communicating
- ❑ **Restless, active, non aggressive behaviour** — for example pacing and aimless wandering, pushing objects around, collecting things, monotonous repetitive activity/movements
- ❑ **Physically aggressive behaviour** — for example hitting, biting, scratching, pushing
- ❑ **Verbally aggressive behaviour** — for example cursing, screaming, threatening, insulting loudly
- ❑ **Verbally non aggressive behaviour** — for example repetitive questions, repetitive sentences, shouting, moaning, screaming, making noises
- ❑ **Other behaviour** — for example uninhibited

2. *Exactly how is the resident behaving in the challenging situation?*

Please take notes (cues) ____

____

3. *When did the behaviour occur for the first time?* ❑ **Unknown**

Please write down period of time/moment: ____ ❑ Unclear

4. *Did a particular event take place shortly before the behaviour occurred for the first time?* ❑ **Unknown**

❑ Move in ❑ New room neighbor ❑ Illness ❑ Change in nursing team ❑ Unclear

❑ Stay in hospital ❑ Change of medications ❑ Others, please note: ____________________

5. *At what time (daytime/night-time) does the behaviour <u>usually</u> occur (in the last fortnight)?*

1am	2	3	4	5	6	7	8	9	10	11	12	1pm	2	3	4	5	6	7	8	9	10	11	12

Remarks on the time: ____

Remarks: ____ ❑ Unclear

6. *For how long does the behaviour <u>usually</u> last (in the last fortnight)?*

❑ Short period of time, a few minutes ❑ _____ hours per day ❑ Nearly the whole time ❑ Unclear

Remarks: ____

7. *How often does the behaviour <u>usually</u> occur (in the last fortnight)?*

❑ Rare (less than once a week) ❑ Once a week ❑ Several times per week ❑ Once a day ❑ Unclear

❑ Several times per day ❑ Several times an hour ❑ Continuously/always

Remarks: ____

Copyright: M. Halek (2010), German Center for Neurodegenerative Diseases (DZNE), Witten

8. *Where does the challenging behaviour usually happen (in the last fortnight)?*

Place: ________________ ❑ Unclear

Remarks: ________________

9. *Who is usually present while the challenging behaviour occurs (in the last fortnight)?* ❑ No other people

❑ Nurses/staff (who exactly?) ________ ❑ Doctors (who exactly?) ________ ❑ Unclear

❑ Other residents (who exactly?) ________ ❑ Others (who exactly?) ________

❑ Friends/Family (who exactly?) ________

Remarks: ________________

10. *Are there certain situations in which the behaviour usually occurs (in the last fortnight)?*

Yes, which ones?: ________________ ❑ No ❑ Unclear

11. *Which emotions or reactions does the behaviour cause in the people involved?*

❑ Please take notes: ________________ ❑ Unclear

12. *What consequences/effects do these emotions/reactions have on the resident´s behaviour?*

❑ Non ❑ Unclear

❑ Positive consequences: Behaviour is influenced in a positive way

❑ Negative consequences: Behaviour is influenced in a negative way

Remarks: ________________

13. *Are there any signs, that the behaviour shown by the resident is irksome or unpleasant?*

For the resident	❑ No	❑ Unclear	❑ Yes, in what respect: ________
For other residents	❑ No	❑ Unclear	❑ Yes, in what respect: ________
For the staff	❑ No		❑ Yes, in what respect: ________

14. Are there any signs, that the behaviour shown by the resident has an impact on the issue of safety?

Resident (puts himself/herself at risk)	❑ No	❑ Unclear	❑ Yes, in what respect: ________
Other Residents (puts other residents at risk)	❑ No	❑ Unclear	❑ Yes, in what respect: ________
Staff (puts staff at risk)	❑ No		❑ Yes, in what respect: ________

Copyright: M. Halek (2010), German Center for Neurodegenerative Diseases (DZNE), Witten

Area I: Cognitive status

1. *Can a delirium/ an acute clouding of consciousness be excluded?* ❑ Yes ❑ No ❑ Unclear

What has to be done?
- ○ Clarification necessary
- ○ Interventions necessary
- ○ Stays important

2. *Has the resident been diagnosed with dementia?* ❑ No ❑ Unclear

Yes, by whom:________________, when:__________ Type of dementia:__________

- ○ Clarification necessary
- ○ Interventions necessary
- ○ Stays important

3. At what stage of dementia is the resident?

Stage: ________________

According to:________________

❑ If unknown please assess here (according to…)

Severity	Cognition	Mode of living	Mini-Mental State Examination
o mild	Solving of complicated tasks is not possible	Limited but possible	Less than 23-24 points
o moderate	Solving of simple tasks partially possible	Depends on help	Less than 20 points
o severe	Threads are not comprehensible anymore	Independent living is impossible	Less than 10 points

- ○ Clarification necessary
- ○ Interventions necessary
- ○ Stays important

4. *Which of the resident´s memory functions are still intact?*

a. *Does s/he still remember important events from her/his past?* ❑ Unclear

❑ Yes, most of the time/nearly everything
❑ Parts
❑ Only a few things/seldom
❑ Nearly nothing

❑ Ability to remember is unstable
❑ Ability to remember is stable

Remarks: ________________________________

- ○ Clarification necessary
- ○ Interventions necessary
- ○ Stays important

b. *Can s/he her/his own personal data (name, age marital status, profession etc.)?* ❑ Unclear

❑ Mostly unrestricted
❑ Partly restricted
❑ Strongly restricted
❑ Nearly nothing

❑ Ability to remember is unstable
❑ Ability to remember is stable

Remarks: ________________________________

- ○ Clarification necessary
- ○ Interventions necessary
- ○ Stays important

Copyright: M. Halek (2010), German Center for Neurodegenerative Diseases (DZNE), Witten

Area I: Cognitive status

4. *Which of the resident´s memory functions are still intact?*

Assessment	What has to be done?
**c. *Is s/he aware of her/his present situation (place of living, life circumstances, illnesses etc.)?*** ❑ Unclear<br>❑ Yes, most of the time/nearly everything<br>❑ Parts<br>❑ Only a few things/seldom ❑ Ability to remember is unstable<br>❑ Nearly nothing ❑ Ability to remember is stable<br>Remarks:	○ Clarification necessary<br>○ Interventions necessary<br>○ Stays important
**d. *Does s/he find her/his way around in the rooms that are important for her/his everyday life? (e.g. her/his private room, in the residential areas, in the home, outside the home)*** ❑ Unclear<br>❑ Mostly unrestricted<br>❑ Partly restricted<br>❑ Strongly restricted ❑ Sense of orientation is unstable<br>❑ Nearly nothing ❑ Sense of orientation is stable<br>Remarks:	○ Clarification necessary<br>○ Interventions necessary<br>○ Stays important
**e. *Does s/he still have sense of time (time of day, time of weeks, seasons etc.)?*** ❑ Unclear<br>❑ Mostly unrestricted<br>❑ Partly restricted<br>❑ Strongly restricted ❑ Sense of time is unstable<br>❑ Nearly nothing ❑ Sense of time is stable<br>Remarks:	○ Clarification necessary<br>○ Interventions necessary<br>○ Stays important
**f. *Is s/he able to complete an activity (brushing teeth, eating her/his meal etc.)?*** ❑ Unclear<br>❑ Mostly unrestricted<br>❑ Partly restricted<br>❑ Strongly restricted ❑ Ability is unstable<br>❑ Nearly nothing ❑ Ability is stable<br>Remarks:	○ Clarification necessary<br>○ Interventions necessary<br>○ Stays important

Copyright: M. Halek (2010), German Center for Neurodegenerative Diseases (DZNE), Witten

Area I: Cognitive status

4. *Which of the resident´s memory functions are still intact?*

What has to be done?

g. *Does she/he recognize important items of everyday life (agnosia)?* ❑ Unclear

- ❑ Mostly unrestricted
- ❑ Partly restricted
- ❑ Strongly restricted
- ❑ Nearly nothing

- ❑ Ability is unstable
- ❑ Ability is stable

Remarks: ____________________

- ○ Clarification necessary
- ○ Interventions necessary
- ○ Stays important

h. *Does s/he remember information, received a day or less beforehand?* ❑ Unclear

- ❑ Mostly unrestricted
- ❑ Partly restricted
- ❑ Strongly restricted
- ❑ Nearly nothing

- ❑ Ability is unstable
- ❑ Ability is stable

Remarks: ____________________

- ○ Clarification necessary
- ○ Interventions necessary
- ○ Stays important

Area II: Physical situation and discomfort

5. *Does s/he show any mobility-related impairment?*

**Change of body / positional change**	❑ No limitation	❑ Limitations		❑ Unclear
**Change of place / locomotion**	❑ No limitation	❑ Limitations		❑ Unclear
**Stamina**	❑ No limitation	❑ Limitations		❑ Unclear
**Coordination**	❑ No limitation	❑ Limitations		❑ Unclear
**Movement need**	❑ increased	❑ decreased	❑ Does not fit	❑ Unclear

Other limitations: ____________________

- ○ Clarification necessary
- ○ Interventions necessary
- ○ Stays important

6. *Does she/he have problems in connection with food or fluid intake?*

**Hunger**	❑ Yes	❑ No		❑ Unclear
**Thirst**	❑ Yes	❑ No		❑ Unclear
**Oral health / dental status**	❑ No limitation	❑ Limitations		❑ Unclear
**Ability to swallow**	❑ No limitation	❑ Limitations		❑ Unclear
**Energy requirements**	❑ increased	❑ decreased	❑ Does not fit	❑ Unclear
**Independent food intake**	❑ No limitation	❑ Limitations		❑ Unclear

Other: ____________________

- ○ Clarification necessary
- ○ Interventions necessary
- ○ Stays important

Copyright: M. Halek (2010), German Center for Neurodegenerative Diseases (DZNE), Witten

Area II: Physical situation and discomfort

						What has to be done?
7. Does s/he show limitations with bowel or bladder function?						○ Clarification necessary<br>○ Interventions necessary<br>○ Stays important
**Urge to urinate/urinary retention**	❑ increased		❑ decreased	❑ Does not fit	❑ Unclear	
**Defecation**	❑ Obstipation		❑ Diarrhoea	❑ Does not fit	❑ Unclear	
**Urinary tract infection**	❑ Yes		❑ No		❑ Unclear	
Other: ____						
8. Does s/he have sleep problems?						○ Clarification necessary<br>○ Interventions necessary<br>○ Stays important
**Disturbed nightly sleep**	❑ Yes		❑ No		❑ Unclear	
**Circadian dysryhthmia**	❑ Yes		❑ No		❑ Unclear	
**Sleeps a lot at during the day**	❑ Yes		❑ No		❑ Unclear	
Other sleeping problems: ____						
9. Does s/he have trouble with vital physical functions?						○ Clarification necessary<br>○ Interventions necessary<br>○ Stays important
**Breathing trouble**	❑ Yes		❑ No		❑ Unclear	
**Circulatory disorders**	❑ Yes		❑ No		❑ Unclear	
**Blood pressure**	❑ high	❑ low	❑ normal	❑ Does not fit	❑ Unclear	
**Fever**	❑ Yes		❑ No			
Other: ____						
10. Are there signs of a depression?	❑ Yes		❑ No		❑ Unclear	○ Clarification necessary<br>○ Interventions necessary<br>○ Stays important
11. Is it possible that she/he is in pain?	❑ Yes, see assessment according to			❑ No	❑ Unclear	○ Clarification necessary<br>○ Interventions necessary<br>○ Stays important
12. Is it possible that she/he has delusions or hallucinations?						○ Clarification necessary<br>○ Interventions necessary<br>○ Stays important
Delusions	❑ Yes		❑ No		❑ Unclear	
Hallucinations	❑ Yes		❑ No		❑ Unclear	
13. Does s/he take drugs, which causes adverse side effects?		❑ No		❑ Unclear		○ Clarification necessary<br>○ Interventions necessary<br>○ Stays important
❑ Yes, which medication and which side effects: ____						
14. Does s/he have other significant illnesses, physical impairment or discomfort?			❑ No		❑ Unclear	○ Clarification necessary<br>○ Interventions necessary<br>○ Stays important
❑ Yes, please note: ____						

Copyright: M. Halek (2010), German Center for Neurodegenerative Diseases (DZNE), Witten

Area III: Independence in everyday-life

15. *Is s/he emotionally burdened/stressed by being limited in self-maintenance/ self-care ability for daily life activities/everyday situations?*

Dependency with movement/locomotion	❑ No burden/stress	❑ Burden/stress	❑ Unclear
Dependency with physical care	❑ No burden/stress	❑ Burden/stress	❑ Unclear
Dependency with getting dresses	❑ No burden/stress	❑ Burden/stress	❑ Unclear
Dependency with fluid/food intake	❑ No burden/stress	❑ Burden/stress	❑ Unclear
Dependency with execration	❑ No burden/stress	❑ Burden/stress	❑ Unclear

Other (for example getting in contact with others): ____________________

○ Clarification necessary
○ Interventions necessary
○ Stays important

16. ***Does nursing-assistance in everyday life activities/situations cause stress or burden for the resident?***

❑ Yes, which: ____________________ ❑ No ❑ Unclear

○ Clarification necessary
○ Interventions necessary
○ Stays important

Summary

A 1. ***Can the challenging behaviour be explained by the type or stage of dementia?*** ❑ **No**

❑ **Probably yes** please take down possible correlations ____________________

____

____

❑ To be taken into account in interactions with the resident ❑ Plan interventions:

A 2. ***Can the identified cognitive impairments explain the challenging behaviour?*** ❑ **No**

❑ Events form the past
❑ Present living situation
❑ Sense of time
❑ Recognizing important everyday items
❑ Information about oneself
❑ Orientation in important rooms
❑ Complete activities
❑ Remembering information received a day or less beforehand

❑ **Probably yes** please take down possible correlations ____________________

____

____

❑ To be taken into account in interactions with the resident ❑ Plan interventions:

Copyright: M. Halek (2010), German Center for Neurodegenerative Diseases (DZNE), Witten

A 3.*Can the identified physical impairment or discomfort somehow related to the challenging behaviour?* ❑ **No**

❑ Mobility ❑ Fluid and food intake ❑ Excretion functions
❑ Sleep ❑ Vital physical functions ❑ Depression
❑ Pain ❑ Delusions/hallucinations ❑ Medication with adverse side-effects
❑ Other illnesses/physical impairments
❑ **Probably yes** please take down possible correlations

❑ To be taken into account in interactions with the resident ❑ Plan interventions:

A 4. *Can the identified stressful/burdening dependencies in everyday-life activities have provoked the challenging behaviour?* ❑ **No**

❑ Emotional burden/stress by care dependencies ❑ Emotional burden/stress by care interventions
❑ **Probably yes** please take down possible correlations

❑ To be taken into account in interactions with the resident ❑ Plan interventions:

Copyright: M. Halek (2010), German Center for Neurodegenerative Diseases (DZNE), Witten

	What has to be done?
**1. *Does the resident hear properly?***<br>❑ Without difficulties and without aids ❑ Without troubles but with aids ❑ Unclear<br>❑ With limitations ❑ Without aids ❑ With aids<br>Remarks:	○ Clarification necessary<br>○ Interventions necessary<br>○ Stays important
**2. *Can the resident see properly?***<br>❑ Without difficulties and without aids ❑ Without troubles but with aids ❑ Unclear<br>❑ With limitations ❑ Without aids ❑ With aids<br>Remarks:	○ Clarification necessary<br>○ Interventions necessary<br>○ Stays important
**3. *In what language can verbal communication with the resident take place?***<br>❑ English ❑ Other languages: ❑ Unclear<br>Remarks:	○ Clarification necessary<br>○ Interventions necessary<br>○ Stays important
**4. *Is the resident´s verbal/nonverbal communication understandable to others?*** ❑ Unclear<br>Verbal: ❑ always ❑ sometimes ❑ never Remarks:<br>Nonverbal: ❑ always ❑ sometimes ❑ never Remarks:	○ Clarification necessary<br>○ Interventions necessary<br>○ Stays important
**5. *If verbal communication with the resident is possible, in what form does it take place (quality of verbal communication)?***<br>❑ Speaks in coherent sentences ❑ Uses single, but understandable words ❑ Unclear<br>❑ Uses word which do not make sense (for other people) ❑ Uses only sounds<br>Remarks:	○ Clarification necessary<br>○ Interventions necessary<br>○ Stays important
**6. *Does the resident comprehend verbal / non verbal utterances made by others?***<br>**Comprehends verbal communication** ❑ Unclear<br>❑ always ❑ sometimes ❑ never<br>❑ Reacts when being addressed ❑ Does not react when being addressed<br>Remarks:<br>**Comprehends non verbal communication**<br>❑ always ❑ sometimes ❑ never<br>❑ Reacts when being addressed ❑ Does not react when being addressed<br>Remarks:	○ Clarification necessary<br>○ Interventions necessary<br>○ Stays important

Copyright: M. Halek (2010), German Center for Neurodegenerative Diseases (DZNE), Witten

7. ***Can the resident comprehend written messages?***<br>❑ always ❑ sometimes ❑ never ❑ Unclear<br>Remarks: ___	**What has to be done?**<br>○ Clarification necessary<br>○ Interventions necessary<br>○ Stays important
8. ***Does the resident clearly utter her/his own wishes and needs (verbally/non-verbally)?***<br>❑ Usually clearly utters personal wishes/needs ❑ Seldom utters clearly personal wishes/needs ❑ Unclear<br>❑ Cannot clearly utter personal wishes/needs<br>Remarks: ___	○ Clarification necessary<br>○ Interventions necessary<br>○ Stays important
9. ***Is the resident capable of making contact to others?***<br>❑ Yes ❑ No ❑ Unclear<br>Remarks: ___	○ Clarification necessary<br>○ Interventions necessary<br>○ Stays important

Summary

B 1. *Is it possible that the identified comprehension/ communication difficulties have triggered/ provoked the challenging behaviour?* ❑ No

❑ Hearing ❑ Seeing well ❑ Language of communication
❑ Comprehensibility of speech ❑ Quality of verbal communication ❑ Understands verbal communication
❑ Resident understands written language ❑ Contact to others ❑ Can communicate personal wishes/desires
❑ **Probably yes** please take down possible correlations ___

___

___

❑ To be taken into account in interactions with the resident ❑ Plan interventions:

B 2. *Is it possible for the behaviour itself to present a form of communication and to explain the behaviour accordingly?* ❑ No

❑ **Probably yes** please take down possible correlations ___

___

___

❑ To be taken into account in interactions with the resident ❑ Plan interventions:

Copyright: M. Halek (2010), German Center for Neurodegenerative Diseases (DZNE), Witten

	**What has to be done?**
**1.** ***How can the resident´s character/personality prior to the onset of dementia be described best?*** *(e.g. ten years before the onset of dementia - more than one answer is possible)*<br>Please mark the tendencies with x ❑ Unclear<br>Emotionally tough, robust ◄——► emotionally sensitive, vulnerable<br>Extroverted, sociable ◄——► loner, introverted<br>Willing to try something new ◄——► conservative<br>Trustful, good-natured, ready to help ◄——► suspicious, egocentric<br>Purposeful, reliable, sense of duty ◄——► inattentive, erratic, spontaneous<br>Other: __________<br>❑ Not known/not assessable	○ Clarification necessary<br>○ Interventions necessary<br>○ Stays important
**2.** ***How high has the resident´s stress and frustration tolerance been with regard to strain- and stressful situations?***<br>❑ Stress tolerance at normal level (reacted balanced to stress situations) ❑ Unclear<br>❑ Low stress tolerance (easily stressed)<br>❑ High stress tolerance (could bear a lot of stress)<br>Remarks: __________<br>❑ Not known/ not assessable	○ Clarification necessary<br>○ Interventions necessary<br>○ Stays important
**3.** ***How did the resident usually cope with stressful situations before the onset of dementia (more than one answer is possible)?***<br>❑ Rather active (for example movement/activity like sports, housework, crafting) ❑ Unclear<br>❑ Rather passive (for example relaxing through watching TV, listening to music, reading)<br>❑ Trough contact, talking to others<br>❑ Through being alone, introverted<br>❑ Being afraid, being reserved, unsure of herself/himself, giving up<br>❑ Over active, aggressive, very emotionally, short-tempered<br>Other: __________<br>❑ Not known/not assessable	○ Clarification necessary<br>○ Interventions necessary<br>○ Stays important

Copyright: M. Halek (2010), German Center for Neurodegenerative Diseases (DZNE), Witten

	What has to be done?
**4.** ***Have there been any particular past situations or events which have been strongly threatening to the person or related to negative emotions (incisive events)?***<br>❑ No ❑ Yes, which ones ______<br>______<br>______<br>❑ Not known/not assessable ❑ Unclear	○ Clarification necessary<br>○ Interventions necessary<br>○ Stays important
**5.** ***Have there been any particular pleasant situations or events in the past which have had a positive influence on life or have been associated with strong positive feelings?***<br>❑ No ❑ Yes, which ones ______<br>______<br>______<br>❑ Not known/not assessable ❑ Unclear	○ Clarification necessary<br>○ Interventions necessary<br>○ Stays important
**6.** ***Which leisure-activities have been preferred by the resident prior to her/his dementia (more than one answer is possible)?***<br>❑ Rather passive activities (for example watching TV, reading, listening to music, relaxing) ❑ Unclear<br>❑ Rather active activities (for example sports, gardening, dancing, hiking)<br>❑ Rather creative activities (for example drawing, knitting, tailoring, baking, playing music)<br>❑ Group activities (for example team games, playing cards, bowling, cooking together)<br>❑ Solidarity activities (for example individual sports, taking a stroll, museum visits, reading)<br>❑ Different activities: ______<br>❑ Not known/not assessable	○ Clarification necessary<br>○ Interventions necessary<br>○ Stays important
**7.** ***Which kind of job/housework did the resident do before s/he fell ill (more than one answer is possible)?***<br>Please name the job: ______ ❑ Unclear<br>❑ Physically passive jobs (office work, driver, shop assistant)<br>❑ Physically active jobs (building, farming, housework)<br>❑ Much contact with other people at work<br>❑ Hardly any contact to other people while working	○ Clarification necessary<br>○ Interventions necessary<br>○ Stays important
**8.** ***Has there been a stable daily rhythm or firm daily rituals to which the resident attached particular importance?***<br>❑ No ❑ Yes, which one(s): ______ ❑ Unclear	○ Clarification necessary<br>○ Interventions necessary<br>○ Stays important

Copyright: M. Halek (2010), German Center for Neurodegenerative Diseases (DZNE), Witten

Summary

C 1. ***Can the challenging behaviour be an expression of the resident´s personality?*** ❑ No

❑ Personality before the onset of dementia ❑ Stress tolerance resp. frustration tolerance

❑ Management of stressful situations before the dementia

❑ **Probably yes** please take down possible correlations

❑ To be taken into account in interactions with the resident ❑ Plan interventions:

C 2. ***Can the challenging behaviour be related to past life events or the person´s former lifestyle?*** ❑ No

❑ Events, which were connected with negative emotions or events which were threatening

❑ Events which were associated with positive impact/emotions

❑ Leisure time before the onset of dementia

❑ Occupation ❑ Daily rhythm/daily rituals with special importance

❑ **Probably yes** please take down possible correlations

❑ To be taken into account in interactions with the resident ❑ Plan interventions:

C 3. ***Can the challenging behaviour be a reaction to stress?*** ❑ No

❑ **Probably yes** please take down possible correlations

❑ To be taken into account in interactions with the resident ❑ Plan interventions:

Copyright: M. Halek (2010), German Center for Neurodegenerative Diseases (DZNE), Witten

Question	What has to be done?
**1. *Does the resident convey the impression that s/he is scared?*** ❑ No<br>❑ sometimes ❑ often ❑ Not assessable ❑ Unclear	○ Clarification necessary<br>○ Interventions necessary<br>○ Stays important
**2. *Are there situations/times of the day that trigger states of anxiety?*** ❑ No<br>❑ Yes, which: ____________ ❑ Unclear<br>❑ Not assessable	○ Clarification necessary<br>○ Interventions necessary<br>○ Stays important
**3. *Does the resident convey the impression to be tired or fatigued?*** ❑ No<br>❑ Sometimes, at which times of the day? ❑ Unclear<br>1am | 2 | 3 | 4 | 5 | 6 | 7 | 8 | 9 | 10 | 11 | 12 | 1pm | 2 | 3 | 4 | 5 | 6 | 7 | 8 | 9 | 10 | 11 | 12<br>❑ Often, at which times of the day?<br>1am | 2 | 3 | 4 | 5 | 6 | 7 | 8 | 9 | 10 | 11 | 12 | 1pm | 2 | 3 | 4 | 5 | 6 | 7 | 8 | 9 | 10 | 11 | 12<br>❑ Not assessable	○ Clarification necessary<br>○ Interventions necessary<br>○ Stays important
**4. *Are there certain events/ situations which exhaust or tire out the resident?*** ❑ No<br>❑ Yes, which: ____________ ❑ Unclear<br>❑ Not assessable	○ Clarification necessary<br>○ Interventions necessary<br>○ Stays important
**5. *Does the resident convey the impression of loneliness or isolation?***<br>❑ Sometimes, at which times of the day? ❑ Unclear<br>1am | 2 | 3 | 4 | 5 | 6 | 7 | 8 | 9 | 10 | 11 | 12 | 1pm | 2 | 3 | 4 | 5 | 6 | 7 | 8 | 9 | 10 | 11 | 12<br>❑ Often, at which times of the day?<br>1am | 2 | 3 | 4 | 5 | 6 | 7 | 8 | 9 | 10 | 11 | 12 | 1pm | 2 | 3 | 4 | 5 | 6 | 7 | 8 | 9 | 10 | 11 | 12<br>❑ Not assessable	○ Clarification necessary<br>○ Interventions necessary<br>○ Stays important
**6. *Are there people, who have a closer relationship with the resident?*** ❑ No<br>❑ Yes, which people: ❑ Unclear	○ Clarification necessary<br>○ Interventions necessary<br>○ Stays important

Copyright: M. Halek (2010), German Center for Neurodegenerative Diseases (DZNE), Witten

Question	What has to be done?
7. Do these relationships convey trust, security, physical contact, affection and acceptance to the resident (tick all applicable)?<br>❑ Truthfulness ❑ Safety ❑ Physical contact ❑ Affection ❑ Acceptance ❑ Unclear<br>❑ Other: ________	○ Clarification necessary<br>○ Interventions necessary<br>○ Stays important
8. At what times of the day is the resident left without some kind of occupation – be it care procedures, activity supply, self induced activities or interests, sleep etc)?<br>Monday to Friday<br>1am | 2 | 3 | 4 | 5 | 6 | 7 | 8 | 9 | 10 | 11 | 12 | 1pm | 2 | 3 | 4 | 5 | 6 | 7 | 8 | 9 | 10 | 11 | 12<br>Saturday and Sunday<br>1am | 2 | 3 | 4 | 5 | 6 | 7 | 8 | 9 | 10 | 11 | 12 | 1pm | 2 | 3 | 4 | 5 | 6 | 7 | 8 | 9 | 10 | 11 | 12<br>Remarks on the time: ________	○ Clarification necessary<br>○ Interventions necessary<br>○ Stays important
9. Do the activities suggested to and taken up by the resident meet his/her personal preferences?<br>❑ Yes ❑ No ❑ Sometimes ________ ❑ Unclear	○ Clarification necessary<br>○ Interventions necessary<br>○ Stays important
10. Does the resident convey the impression of being bored most of the day time? ❑ No<br>Sometimes, at which times of the day? ❑ Unclear<br>1am | 2 | 3 | 4 | 5 | 6 | 7 | 8 | 9 | 10 | 11 | 12 | 1pm | 2 | 3 | 4 | 5 | 6 | 7 | 8 | 9 | 10 | 11 | 12<br>❑Or situations: ________ ❑ Not assessable<br>Often, at which times of the day?<br>1am | 2 | 3 | 4 | 5 | 6 | 7 | 8 | 9 | 10 | 11 | 12 | 1pm | 2 | 3 | 4 | 5 | 6 | 7 | 8 | 9 | 10 | 11 | 12<br>❑Or situations: ________ ❑ Not assessable	○ Clarification necessary<br>○ Interventions necessary<br>○ Stays important

Copyright: M. Halek (2010), German Center for Neurodegenerative Diseases (DZNE), Witten

Summary

D 1. ***Can the challenging behaviour be an expression of certain moods and emotions?*** ❑ **No**

❑ Being afraid ❑ Tiredness/exhaustion ❑ Closer relationship to the resident

❑ Relationship show confidence, safety etc. ❑ Times without occupation ❑ Boredom

❑ Occupational activities/leisure time activities/structure of the day do not match the residents personal preferences

❑ **Probably yes** please take down possible correlations

❑ To be taken into account in interactions with the resident ❑ Plan interventions:

D 2. ***Can the challenging behaviour serve as self-stimulation?*** ❑ **No**

❑ **Probably yes** please take down possible correlations

❑ To be taken into account in interactions with the resident ❑ Plan interventions:

Copyright: M. Halek (2010), German Center for Neurodegenerative Diseases (DZNE), Witten

	What has to be done?
**1. *What characterizes the environment in which the resident spends most of her/his time?***	
**a.** *Lighting?* ❑ Unclear<br>❑ dark ❑ bright/harsh<br>❑ A lot of shadows/reflections ❑ Unpleasant/cold light<br>❑ Same lighting throughout the day ❑ Light which cannot be adjusted individually<br>❑ Lighting at night<br>❑ Does not fit ❑ Other:__________	○ Clarification necessary<br>○ Interventions necessary<br>○ Stays important
i. *Noises in the environment?* ❑ Unclear<br>❑ noisy ❑ quiet<br>❑ Many different noises, permanent music, telephone, signal of nurse call button etc.<br>❑ Does not fit ❑ Other:__________	○ Clarification necessary<br>○ Interventions necessary<br>○ Stays important
**b.** *Smells/odours?* ❑ Unclear<br>❑ Unpleasant smells ❑ Many different smells<br>❑ No homely/known smells<br>❑ Does not fit ❑ Other:__________	○ Clarification necessary<br>○ Interventions necessary<br>○ Stays important
c. *The furnishing / the equipment?* ❑ Unclear<br>❑ Not personal ❑ Only practical, not home like<br>❑ Uncomfortable/unfriendly<br>❑ Does not fit ❑ Other:__________	○ Clarification necessary<br>○ Interventions necessary<br>○ Stays important
**d.** *Sense of safety/familiarity?* ❑ Unclear<br>❑ Strange, unknown surroundings ❑ People I do not recognize<br>❑ No obvious orientation signs/no orientation signs that suit cognitive abilities<br>❑ Danger of injuries/falls ❑ Danger of getting lost<br>❑ Restricted movement<br>❑ Does not fit ❑ Other:__________	○ Clarification necessary<br>○ Interventions necessary<br>○ Stays important

Copyright: M. Halek (2010), German Center for Neurodegenerative Diseases (DZNE), Witten

	**What has to be done?**
**1. *What characterizes the environment in which the resident spends most of her/his time?*** ***a.*** *Sense of privacy?* ❑ Unclear ❑ No possibility to be on ones´ own/to retreat ❑ No protected privacy ❑ No possibility to fulfill private desires ❑ No possibility for ones´ own time structure ❑ No individual daily rhythm ❑ Does not fit ❑ Other: ________	○ Clarification necessary ○ Interventions necessary ○ Stays important
b. *Positive stimuli/stimulation (persons or objects)?* ❑ Unclear ❑ A little/few stimuli, boring surroundings (understimulation) ❑ Lots of stimuli (overstimulation) ❑ Does not fit ❑ Other: ________	○ Clarification necessary ○ Interventions necessary ○ Stays important
**2. *Does the resident´s environment provide enough chances for making contact with others?*** ❑ Yes ❑ No ❑ Unclear ❑ No possibility to get in touch without help ❑ Long time intervals without verbal stimulation ❑ No persons who fit the resident´s communication interests and levels ❑ Other: ________	○ Clarification necessary ○ Interventions necessary ○ Stays important
**3. *Does the resident prefer certain carer/ caregivers as significant others (closer relationship, easier intercourse, more profound quality of relationship, empathy, gender)?*** ❑ Yes: Please take notes (cues) ________	○ Clarification necessary ○ Interventions necessary ○ Stays important
**4. *How long is an individual caregiver responsible for a certain resident?*** ❑ Unclear ❑ Same caregiver for the week including weekend in the ❑ Early day shift ❑ Late day shift ❑ Same caregiver throughout the week excluding weekend in the ❑ Early day shift ❑ Late day shift ❑ Same caregiver at least three days a week in the ❑ Early day shift ❑ Late day shift ❑ Different/new caregiver every day in the ❑ Early day shift ❑ Late day shift ❑ Other: ________	○ Clarification necessary ○ Interventions necessary ○ Stays important

Copyright: M. Halek (2010), German Center for Neurodegenerative Diseases (DZNE), Witten

Summary

E 1. ***Can the challenging behaviour have to do with certain environmental characteristics?*** ❑ **No**

❑ Lighting ❑ Noise in the surroundings ❑ Smells

❑ Furnishing ❑ Privacy ❑ Getting in touch with others ❑ Positive stimuli/stimulations

❑ **Probably yes** please take down possible correlations

❑ To be taken into account in interactions with the resident ❑ Plan interventions:

E 2. ***Can the challenging behaviour have to do with a lacking sense of security and familiarity?*** ❑ **No**

❑ **Probably yes** please take down possible correlations

❑ To be taken into account in interactions with the resident ❑ Plan interventions:

E 3. ***Can staff structure have an impact on the challenging behaviour?*** ❑ **No**

❑ Preference of caregiver ❑ As a primary caregiver ❑ Continuity of primary caregivers

❑ **Probably yes** please take down possible correlations

❑ To be taken into account in interactions with the resident ❑ Plan interventions:

Copyright: M. Halek (2010), German Center for Neurodegenerative Diseases (DZNE), Witten
