# Supplementary material for: Development and evaluation of the content validity, practicability and feasibility of the Innovative dementia-oriented Assessment system for challenging behaviour in residents with dementia
Source: BMC Health Serv Res. 2017 Aug 14;17:554. doi: 10.1186/s12913-017-2469-8 (PMC5557472; doi:10.1186/s12913-017-2469-8)
Supplement: Supplementary file 2 — Translation process of the German IdA ® into English (IdA-E®) using a forward and backward translation process. (PDF 516 kb) [file 12913_2017_2469_MOESM2_ESM.pdf]

DZNE
Deutsches Zentrum für Neurodegenerative Erkrankungen
in der Helmholtz-Gemeinschaft

INTERDEM
Early detection and timely INTERvention in DEMentia

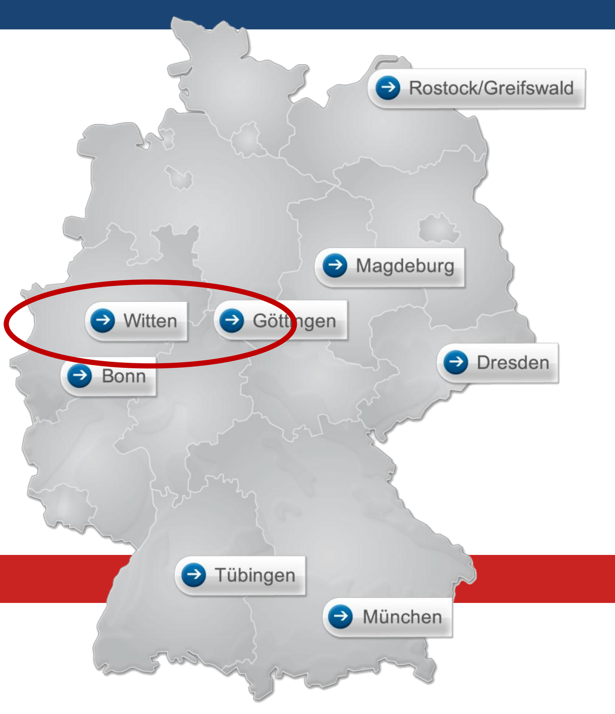

English translation of an assessment for identifying triggers and causes of dementia related challenging behaviour - IdA

Daniela Holle, Matthias Zischka, Margareta Halek

Email: Daniela.Holle@dzne.de

1. Background

- The Innovative Dementia-Oriented Assessment tool (IdA) guides nursing staff systematically through the process of identifying possible triggers and causes of challenging behavior of people with dementia (see Figure 1).
- IdA was developed on the basis of an international literature study and the need-driven-dementia-compromised-behavior model (Kolanowski 1999).
- It was tested in relation to its practicability, content validity and construct validity in different nursing homes in Germany (Halek 2010).
- IdA is recommended as a guideline for team meetings and dementia-specific case conferences (Halek & Bartholomeyczik 2009).
- Use of IdA was limited to the German speaking context because of its restriction to the German language.

2. Aim

- Translate of IdA into the English language as a first step to make IdA available for its use in English speaking countries.

Innovative Dementia Orientated Assessment tool (IdA)

Step I: Assessment of challenging behaviour

A. Description of behaviour (4 items)
B. Frequency of behaviour (3 items)
C. Context of behaviour (3 items)
D. Consequences of behaviour (4 items)

Step II: Analyse of potential triggers of challenging behaviour

E. State of health & independence in every day life (16 items)
F. Communication (9 items)
G. Personality & life style before the dementia (8 items)
H. Moods & emotions (10 items)
I. Environmental influences (5 items)

Figure 1: Innovative Dementia Orientated Assessment tool

3. Method

- Translation of IdA was carried out according to the translation process of ISPOR (Wild, Grove et al. 2005) (see Figure 2).
- First IdA was translated into the target language (English) and subsequently translated back into the original language (German).
- The instrument was checked by two registered nurses from England (Great Britain) for its comprehensibility and conceptual similarities (cognitive debriefing).

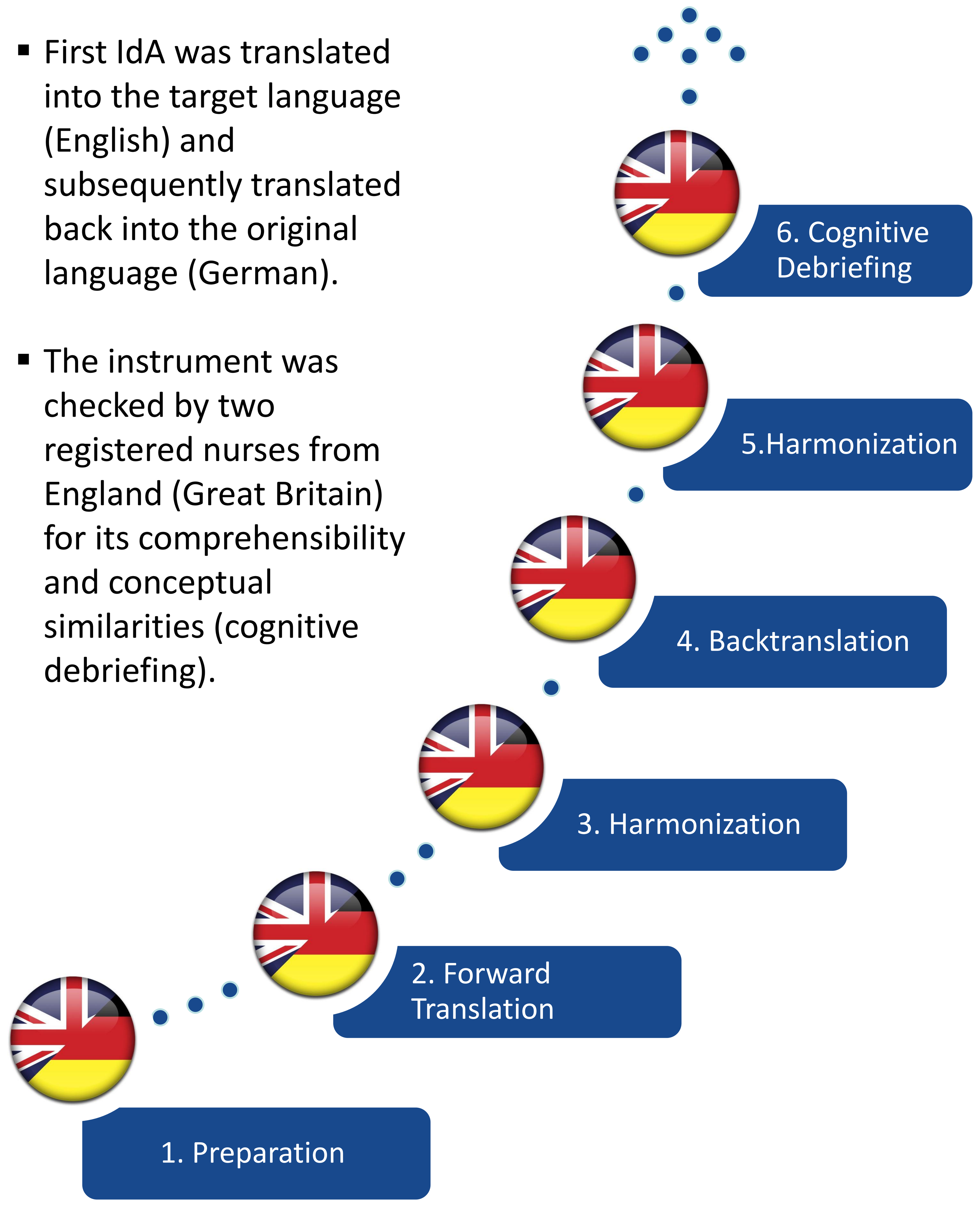

Figure 2: Translation process of IdA

4. Results

- An English version of IdA is now available for use in nursing research and nursing practice (see Figure 3).
- It was of great importance that translators were informed about the underlying concepts of the assessment and that each question of IdA was described in detail in a manual before translation took place
- A further advantage was that all translators had experiences in the field of nursing and dementia.
- The harmonization process was a time consuming step and it tool longer than expected.

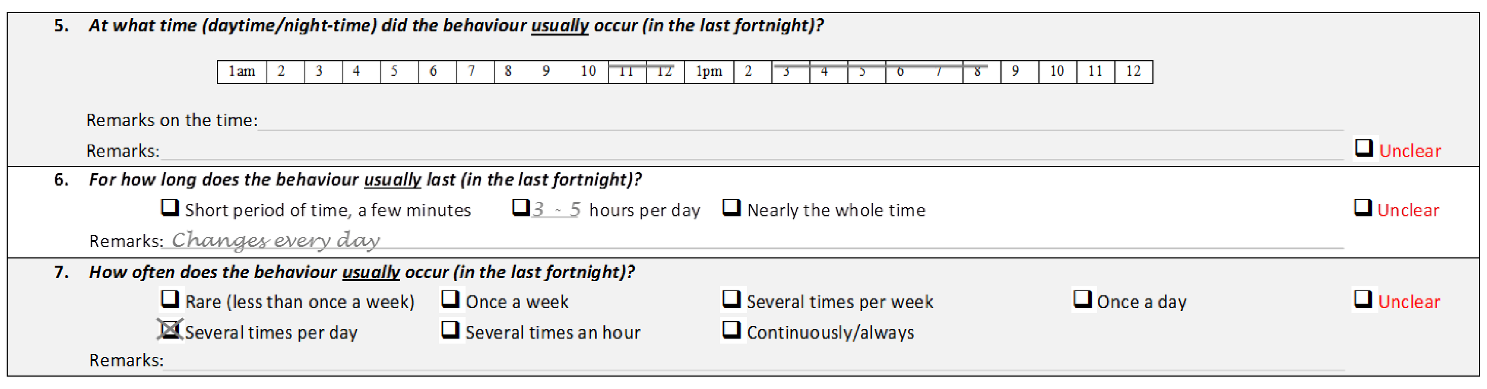

5. *At what time (daytime/night-time) did the behaviour usually occur (in the last fortnight)?*

1am	2	3	4	5	6	7	8	9	10	11	12	1pm	2	3	4	5	6	7	8	9	10	11	12

Remarks on the time:
Remarks: ❑ Unclear

6. *For how long does the behaviour usually last (in the last fortnight)?*
❑ Short period of time, a few minutes ❑ 3 - 5 hours per day ❑ Nearly the whole time ❑ Unclear
Remarks: Changes every day

7. *How often does the behaviour usually occur (in the last fortnight)?*
❑ Rare (less than once a week) ❑ Once a week ❑ Several times per week ❑ Once a day ❑ Unclear
☒ Several times per day ❑ Several times an hour ❑ Continuously/always
Remarks:

Figure 3: Extract of English version of IdA

5. Discussion

- Translation of IdA into English has to be understood as a first step within the adaptation process of IdA for an English-speaking country. Further studies are needed to pilot IdA within the specific context of the target population and to test its linguistic appropriateness as well as its face and content validity. In addition, psychometric testings are needed that evaluate the scientific merit of IdA in the target country.

References:
- Halek M. (2010). Entwicklung und Testung eines strukturierten Assessmentbogens zur Erfassung der Auslöser für herausforderndes Verhalten von Menschen mit Demenz in der stationären Altenhilfe. Fakultät für Medizin, Institut für Pflegewissenschaft. Witten, Witten/ Herdecke.
- Halek M. & Bartholomeyczik S. (2009). 3.4 Assessmentinstrument für die verstehende Diagnostik bei Demenz: Innovatives demenzorientiertes Assessmentsystem (IdA). Assessmentinstrumente in der Pflege. Bartholomeyczik S. & Halek. M. Hannover, Schlütersche Verlagsgesellschaft mbH & Co. KG: 94-104.
- Kolanowski A. M. (1999). "An overview of the Need-Driven Dementia-Compromised Behavior Model." Journal of Gerontological Nursing **25**(9): 7-9.
- Wild D, Grove A, et al. (2005). "Principles of Good Practice for the Translation and Cultural Adaptation Process for Patient-Reported Outcomes (PRO) Measures: Report of the ISPOR Task Force for Translation and Cultural Adaptation." Value in Health **8**(2): 94-104.
